# Dealing with the Evolutionary Downside of CRISPR Immunity: Bacteria and Beneficial Plasmids

**DOI:** 10.1371/journal.pgen.1003844

**Published:** 2013-09-26

**Authors:** Wenyan Jiang, Inbal Maniv, Fawaz Arain, Yaying Wang, Bruce R. Levin, Luciano A. Marraffini

**Affiliations:** 1 Laboratory of Bacteriology, The Rockefeller University, New York, New York, United States of America; 2 Department of Biology, Emory University, Atlanta, Georgia, United States of America; Université Paris Descartes, France

## Abstract

The immune systems that protect organisms from infectious agents invariably have a cost for the host. In bacteria and archaea CRISPR-Cas loci can serve as adaptive immune systems that protect these microbes from infectiously transmitted DNAs. When those DNAs are borne by lytic viruses (phages), this protection can provide a considerable advantage. CRISPR-Cas immunity can also prevent cells from acquiring plasmids and free DNA bearing genes that increase their fitness. Here, we use a combination of experiments and mathematical-computer simulation models to explore this downside of CRISPR-Cas immunity and its implications for the maintenance of CRISPR-Cas loci in microbial populations. We analyzed the conjugational transfer of the staphylococcal plasmid pG0400 into *Staphylococcus epidermidis* RP62a recipients that bear a CRISPR-Cas locus targeting this plasmid. Contrary to what is anticipated for lytic phages, which evade CRISPR by mutations in the target region, the evasion of CRISPR immunity by plasmids occurs at the level of the host through loss of functional CRISPR-Cas immunity. The results of our experiments and models indicate that more than 10^−4^ of the cells in CRISPR-Cas positive populations are defective or deleted for the CRISPR-Cas region and thereby able to receive and carry the plasmid. Most intriguingly, the loss of CRISPR function even by large deletions can have little or no fitness cost in vitro. These theoretical and experimental results can account for the considerable variation in the existence, number and function of CRISPR-Cas loci within and between bacterial species. We postulate that as a consequence of the opposing positive and negative selection for immunity, CRISPR-Cas systems are in a continuous state of flux. They are lost when they bear immunity to laterally transferred beneficial genes, re-acquired by horizontal gene transfer, and ascend in environments where phage are a major source of mortality.

## Author Summary

In addition to the virtue of protecting archaea and bacteria from the ravages of lethal viruses (phage), the immunity generated by the CRISPR-Cas systems have an evolutionary downside; they can prevent the acquisition of genes and genetic elements required for the adaptation and even the survival of these microbes. Using mathematical models and experiments with *Staphylococcus epidermidis* and the staphylococcal conjugative plasmid pG0400, we explore how bacteria deal with this evolutionary downside of CRISPR-Cas immunity. Although there are mechanisms by which immune populations of bacteria can acquire essential plasmids without the loss of CRISPR-Cas immunity, the results of our conjugation and fitness cost experiments suggest the most likely mechanism is the deactivation and deletion of this region. These results provide an explanation for the considerable variation in the existence, number and function of CRISPR-Cas within and between species of microbes. Along with other observations our work also suggests that the CRISPR-Cas loci are in a continuous state of flux: acquired by horizontal gene transfer, ascend when populations are confronted with phage and are rapidly lost when infectiously transmitted genes and genetic elements are required for the adaptation and survival of the population.

## Introduction

Clustered, Regularly Interspaced Short Palindromic Repeat (CRISPR) loci and their associated proteins (Cas) have been found in the genomes of about 90% of archaea and about 50% of eubacteria [1], [2], [3]. In these loci, repeats are separated by short sequences of DNA (25–70 base pair-long), known as “spacers”, most of which are homologous to regions in the genome of the viruses (phages) that attack these microbes [4], [5], [6]. This observation led to the suggestion that the CRISPR-Cas loci may be part of an immune system that defends archaea and bacteria from phage infection, a hypothesis that has been supported by experimental studies [7], [8], [9]. Immunity is achieved by the transcription of CRISPR spacer sequences into small antisense CRISPR RNAs (crRNAs) that act as guides for a crRNA-Cas ribonucleoprotein complex [10], [11], that is ultimately responsible for the sequence-specific identification and destruction of the invader [12], [13]. Depending on the *cas* gene content, CRISPR-Cas systems can be classified into three types and different subtypes: I-A to F, II-A and B, and III-A and B [14]. CRISPR-Cas loci have the ability of acquiring new spacer sequences derived from the invading genome [8], [15], [16], [17], [18], [19], therefore the spacer content of a CRISPR-Cas system is highly dynamic and constitutes a genetic memory of past infections. The details of how these spacers are acquired from infecting DNAs, however, have not yet been fully elucidated.

Theoretically there are broad conditions where CRISPR-mediated immunity will provide bacteria and archaea an advantage in the presence of lytic phage [20], [21], [22] and it is reasonable to assume that CRISPR-Cas systems become established and are maintained in populations of these microbes by phage-mediated selection. However, while the majority of spacer sequences with matches on genebank target bacteriophage genomes, many spacers match plasmids, other mobile genetic elements and chromosomal regions of bacteria and archaea [23], [24]. Thus, in addition to their role as an anti-viral immune system, CRISPR-Cas loci can constitute a barrier against the horizontal transfer of genes and accessory genetic elements. Indeed, CRISPR interference has been shown experimentally to prevent the acquisition of conjugative plasmids [25], integrative conjugative elements [26] and environmental DNA by natural transformation [27] [28].

In principle, accessory genetic elements and foreign genes can engender a fitness burden on their host bacteria and thereby serve as a selective force for the maintenance of CRISPR immunity against these elements [21]. On the other hand, plasmids and foreign genes can also provide bacteria and archaea with a substantial fitness advantage. In fact, much of the adaptation of bacteria to their environment is through the acquisition of DNA via horizontal transfer of genes and accessory genetic elements [29]. Thus CRISPR-mediated immunity against plasmids, transforming DNA and other mobile genetic elements that carry beneficial genes can be an impediment to the survival of prokaryotes and their adaptation to their environment, i.e. an evolutionary downside of CRISPR-Cas immunity.

The staphylococci are a good example of bacteria that rely on the transfer of accessory genetic elements for their adaptation to their environment. Phages and plasmids provide fundamental routes for the spread of staphylococcal virulence determinants [30], [31]. Pathogenic strains have acquired resistance to all known antibiotics [32], primarily through the acquisition of conjugative plasmids carrying resistance genes [33]. These bacteria also bear CRISPR-Cas systems that provide immunity to plasmids. The best studied example is *Staphylococcus epidermidis* RP62a, a clinical isolate containing a type III-A CRISPR-Cas system with three spacers: one matching all staphylococcal conjugative plasmids sequenced to date, a second matching *S. epidermidis* bacteriophages and a third with no homology in genebank [34]. The first spacer of this CRISPR-Cas system mediates CRISPR immunity against the conjugative transfer of the mupirocin-resistant plasmid pG0400 [25]. This antibiotic is commonly applied intranasally to eliminate staphylococcal carriage before surgery [35], and in response to this prophylactic measure staphylococci become resistant through the conjugative spread of mupirocin-resistant conjugative plasmids [36]. Therefore CRISPR immunity would prevent the acquisition of mupirocin resistance by *S. epidermidis* RP62a and compromise the survival of *S. epidermidis* RP62a, and other staphylococci carrying similar CRISPR-Cas systems [37], [38], in hospital or other settings where antibiotics are used.

In this report, we investigate how bacterial populations deal with the evolutionary downside of CRISPR-Cas immunity. To determine the relative likelihood of different mechanisms by which immune CRISPR populations can acquire beneficial plasmids, we performed “offer they can’t refuse” conjugation experiments, where mupirocin-resistant pG0400 plasmids are transferred to mupirocin-sensitive *S. epidermidis* RP62a hosts that require this resistance gene for survival and replication. As observed in analogous experiments with lytic phage [7], [8], [9], [39], [40], plasmids could evade CRISPR-mediated immunity by the introduction of mutations in the target site that eliminate complementarity with the crRNA. Mutant plasmids of this type were previously engineered and shown to avoid CRISPR immunity [25]. Contrary to this expectation, the only evasion mechanisms observed in our experiments occurred at the level of the host, primarily through the inactivation or deletion of the CRISPR-Cas locus or the spacer responsible for this immunity. We performed fluctuation experiments and computer simulations that indicate that mutant cells to which the plasmid is transferred pre-exist and are produced at rates in excess of 10^−4^ per cell per generation. Intriguingly, deletions of the CRISPR-Cas region that encompass substantial fractions of the genome were found to engender little or no fitness costs.

These results provide a possible explanation for the enormous variation in the existence, number and functionality of CRISPR-Cas systems within and between species of bacteria and archaea. We postulate that as a consequence of opposing selection forces the CRISPR-Cas regions are in a continuous state of flux: CRISPR-Cas loci are acquired and spread through horizontal gene transfer [40], [41], [42], ascend in habitats where phage are a major source of mortality and are readily lost or become non-functional in environments where the acquisition of genes and accessory genetic elements from without are critical to or essential for adaptive evolution.

## Results

### Rates of plasmid transfer

To study how bacteria deal with this downside of CRISPR immunity we performed “offer they can’t refuse” plasmid transfer experiments. For this we used *S. epidermidis* RP62a (neomycin-resistant) recipients bearing a CRISPR-Cas system with a spacer matching the *nickase* (*nes*) gene of pG0400, and *S. aureus* RN4220/pG0400 donors harboring the mupirocin-resistant conjugative plasmid targeted by the *S. epidermidis* RP62a CRISPR-Cas system. Cells were grown together on filters, suspended in saline and plated on agar containing both neomycin and mupirocin to select transconjugants, i.e. *S. epidermidis* RP62a cells containing pG0400.

**Figure 1:**
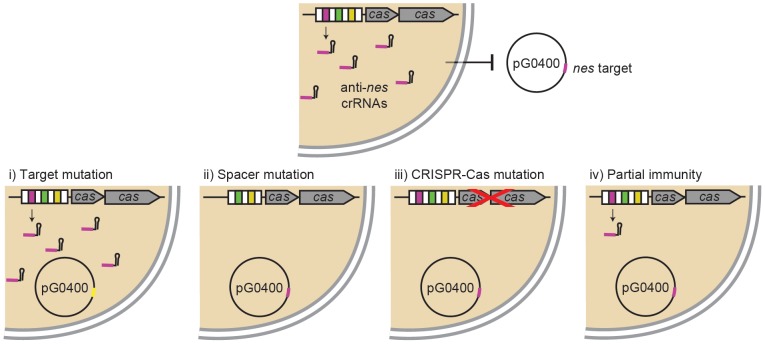
Different possibilities for the transfer of a beneficial plasmid into cells encoding CRISPR immunity against it. *S. epidermidis* RP62a contains a CRISPR-Cas system with a spacer (pink) that produces crRNAs that match and target the *nickase* (*nes*) gene (also in pink) of staphylococcal conjugative plasmids, including pG0400. There are at least four different mechanisms that will allow the transfer of the plasmid in spite of CRISPR immunity: (i) mutation of the plasmid target (yellow), (ii) mutation or deletion of the anti-plasmid spacer, (iii) loss-of-function mutation of the *cas* genes required for immunity or partial or complete deletion of the CRISPR-Cas locus, or (iv) partial immunity that leads to tolerance of the plasmid.

Because in a previous study we used a low level of detection (we could not detect less than 100 transconjugants/ml) and failed to detect transconjugants [25], we decided to elevate our level of detection by increasing 10 fold the number of donors and recipients mated. This allowed us to obtain approximately 15 transconjugants/ml (Table 1). As controls we performed similar transfer experiments with an isogenic recipient lacking the CRISPR repeat-spacer array (Δ*crispr*, [25]), and an *S. aureus* donor carrying pG0400*^mut^*, an otherwise isogenic pG0400 plasmid with 9 silent substitutions in the *nes* target region that eliminate the spacer/target homology [25]. As anticipated from our previous studies [25], the relative rate of plasmid transfer, the quotient of the densities of transconjugants and the product of the densities of donors and recipients [Γ = T/(D*R)] [43], [44], is between 3 and 4 orders of magnitude less for the transfer of pG0400 into *S. epidermidis* RP62a cells than that for the transfer of the plasmid into Δ*crispr* cells or the transfer of pG0400*^mut^* into wild-type hosts (Table 1). These results show that transfer of a plasmid into cells containing a CRISPR-Cas locus that targets this plasmid can occur, presumably by one or more of four distinct mechanisms (Figure 1): (i) mutations in the target region of the plasmid that enable it to evade CRISPR-Cas immunity, (ii) mutations in or deletion of the spacer responsible for the immunity, (iii) the loss of CRISPR-Cas function through mutation or deletion and, (iv) tolerance, where CRISPR-Cas immunity is not absolute (it reduces the rate of receipt of the plasmid but does not prevent its transfer and establishment).

**Table 1:** Relative rate parameters of plasmid transfer.

Plasmid in donor strain(a)	Donor cfu/ml (D)	Recipient *S. epidermidis* strain(b)	Recipient cfu/ml (R)	Trans-conjugants cfu/ml (T)	Frequency of conjugation (T/R)	Γ = T/(D×R)
pG0400	4.8×10^9^	Δ*crispr*	1.3×10^9^	8.0×10^4^	6.2×10^−5^	1.3×10^−14^
	4.1×10^9^		1.1×10^9^	1.9×10^4^	1.7×10^−5^	4.2×10^−15^
pG0400	1.3×10^10^	RP62a	1.5×10^9^	1.4×10^1^	9.3×10^−9^	7.2×10^−19^
	1.6×10^10^		1.0×10^9^	2.3×10^1^	2.3×10^−8^	1.4×10^−18^
pG0400*^mut^*	8.0×10^9^	Δ*crispr*	1.3×10^9^	1.5×10^5^	1.2×10^−4^	1.4×10^−14^
	1.1×10^10^		1.7×10^9^	5.0×10^4^	2.9×10^−5^	2.7×10^−15^
pG0400*^mut^*	1.6×10^10^	RP62a	2.0×10^9^	2.4×10^4^	1.2×10^−5^	7.5×10^−16^
	8.0×10^9^		1.4×10^9^	4.0×10^4^	2.9×10^−5^	3.6×10^−15^

(a) pG0400*^mut^* is a derivative of pG0400 containing 9 silent mutations in the *spc1* CRISPR target.

(b) RP62a is a wild-type strain. Δ*crispr* is an isogenic mutant lacking the CRISPR array.

### Transconjugants are defective for CRISPR-Cas immunity

To determine which of these mechanisms license the transfer and maintenance of the targeted plasmid, we analyzed 111 transconjugants obtained in two transfer experiments. We initiated our molecular genetic analysis by testing for the presence of mutations in the target (scenario i), the seemingly most likely explanation based on results obtained for phages that escape CRISPR immunity [7], [8], [9], [39], [40]. For this we isolated DNA from the transconjugants, amplified the *nes* target region (400 bp) of pG0400 and sequenced the PCR products. In all cases we obtained wild-type sequences, no mutant plasmids were observed (data not shown).

**Figure 2:**
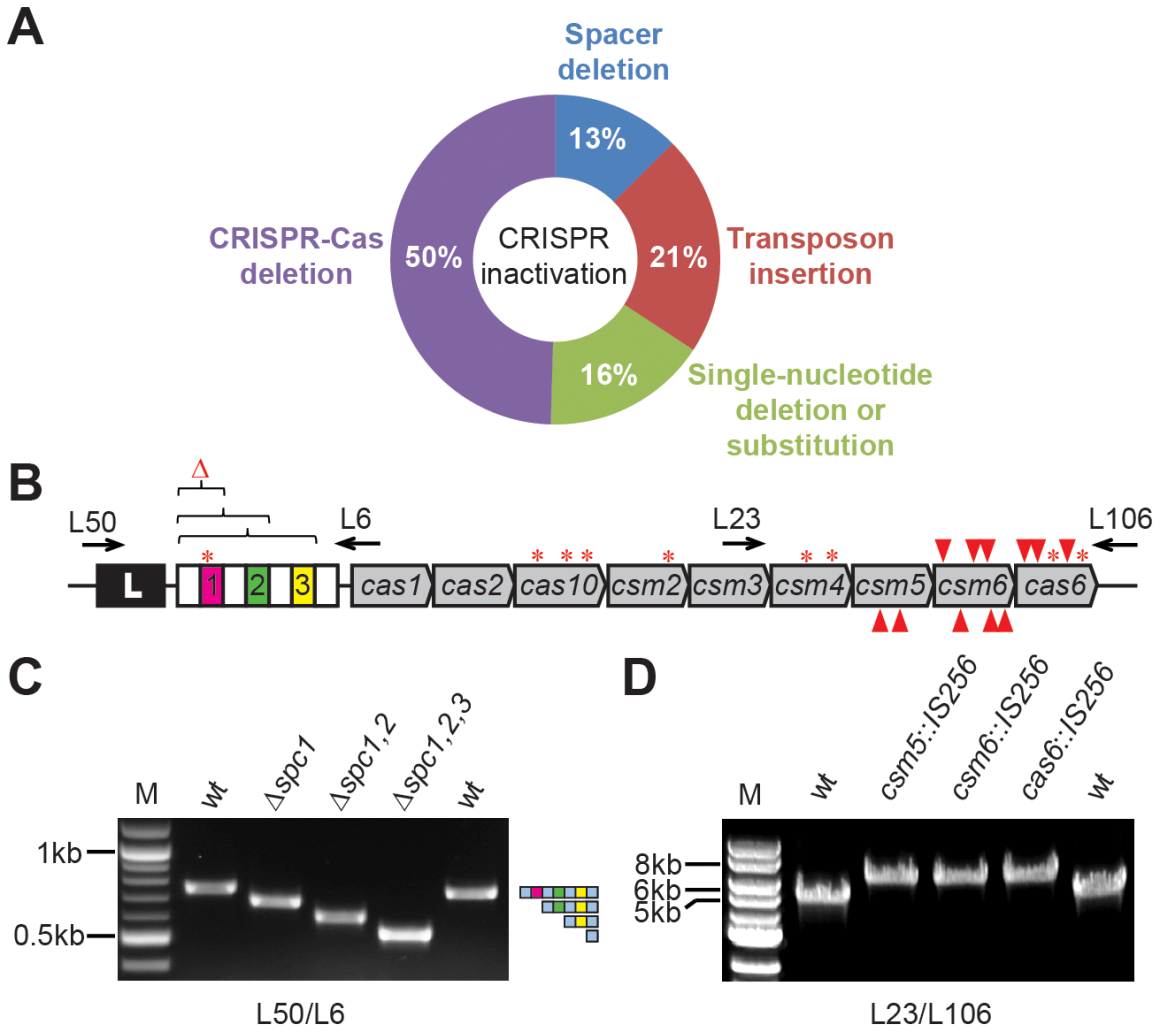
Different mutations eliminate CRISPR immunity against conjugation in *S. epidermidis*. (**A**) Summary of the different mutations found in this study and their proportions. (**B**) Distribution of mutations within the CRISPR-Cas locus. *S. epidermidis* RP62a harbors a CRISPR-Cas system containing four repeats (white boxes), three spacers (colored, numbered boxes) and nine *cas*/*csm* genes. Mutations found in CRISPR escapers include deletions in the repeat-spacer region (brackets), transposon insertions (red arrowheads; top, direct insertion; bottom, inverted) and single nucleotide deletions or substitutions (asterisks). Arrows indicate primers used to analyze transconjugants. (**C**) PCR analysis of the CRISPR array of transconjugants using primers L50/L6. Deletion of 1, 2 and 3 spacers observed in escapers R23, R10 and R2, respectively, is shown. M, DNA marker. *wt*, amplification using wild-type template DNA. (**D**) PCR analysis of the *cas* gene region of escapers using primers L23/L106. IS*256* transposon insertions into *csm5*, *csm6* and *cas6* observed in escapers R60, B15 and R36, respectively, are shown. M, DNA marker. *wt*, amplification using wild-type template DNA.

While we can’t exclude the possibility that CRISPR-Cas immunity to plasmids is somewhat leaky (scenario iv), our molecular genetic analysis of the 111 S. *epidermidis* RP62a/pG0400 transconjugants provided no evidence for this. The only mechanism observed were those of scenario (ii), the mutation or deletion of the spacer sequence matching the plasmid target, and scenario (iii), the presence of non-functional mutations in the CRISPR-Cas region or the complete or partial deletion of the CRISPR-Cas locus (Figure 2A and Table 2).

**Table 2:** Genotype of transconjugants that escape CRISPR immunity.

Transconjugant(a)	Genotype
R1	*csm2*(ΔA297). Frameshift(b)
R2	Δ*spc1,2,3*
R3, R4, R11, R13	*csm6*(617)::IS256*-d* (c)
**R5,** R8, R18, R30, R42	*cas10*(ΔA979). Frameshift
R6	*csm4*(ΔA57). Frameshift
**R7**, R24, R32, R50, R56, R65, R70	Δ2387081–2520252 (IS256)(d)
R9, R12, R25, R27, R39, R41, R45, R59	Δ2470979–2553341 (SERP2409&SERP2493)
R10, R19, R28, R34, R47, R58, R63, B32	Δ*spc1,2*
**R14**	Δ2515497–2581208 (IS431)
R15	Δ2488334–2581208 (IS431)
R16, R33, R37, R40, R49, R54, R55, R57, R61, R62, R67, B3, B6, B9, B18	Δ2399891–2551562 (SERP2353&SERP2491)
R17	*cas6*(193)::IS256*-d*
R20	*spc1*(ΔA1)(e)
R22	Δ2493652–2584194 (OrfX)
R23, R31, R46, B7, B19	Δ*spc1*
R29	Δ2496588–2581208 (IS431)
R35	Δ2295499–2535027 (IS256)
R36, R48	*cas6*(199)::IS256*-d*
R38, R44, R68	*csm6*(1059)::IS256*-d*
R43	*csm4*(G566T; G189V)(f)
R51	Δ2515497–2581208 (IS431)
R52	*cas10*(C1411T; Q471Stop)
R53, R69, B8, B12	*csm2*(ΔA297). Frameshift
**R60**, R64	*csm*5(558)::IS256*-i*
R66	*cas10*(G1017A; W339Stop)
R71	*cas6*(269)::IS256*-d*
B1	Δ2433266–2581208 (IS431)
B2, B10, B11, B21, B25, B27, B31, B40, B42	Δ2437916–2581208 (IS431)
B4, B5, B29, B38	Δ2440320–2582329 (IS256)
B13, B14, B22	*csm6*(1172)::IS256*-i*
B15, B16, B33	*csm6*(953)::IS256*-d*
B17	Δ2359213–2581208 (IS431)
B20	*csm6*(ΔA1238). Frameshift
B23	Δ2401725–2562431 (Tn554)
B24	*csm6*(844)::IS256*-i*
B26	*cas6*(ΔA172). Frameshift
B28	Δ2447777–2581208 (IS431)
B30	Δ2495387–2581208 (IS431)
B34, B35, B36	*csm5*(583)::IS256*-i*
B37	*cas6*(G484T; E162Stop)
**B39**	Δ2274721–2581208 (IS256)
B41	*csm6*(1163)::IS256*-i*

(a) Transconjugants obtained in two independent experiments (R and B) are shown. Transconjugants R21 and R26 are not reported as genotypic analysis indicated these were *S. aureus* RN4220/pG0400 donors that acquired neomycin resistance. Highlighted and underlined are transconjugants that were chosen for the pairwise competition experiments estimating fitness.

(b) The number indicates the adenine residue deleted relative to the start codon of the gene.

(c) The number indicates the position of insertion of the IS*256* element relative to the start codon of the gene. Direct, *d*, or inverted, *i*, refer to the orientation of the transposase gene of the IS*256* element with respect to the direction of transcription of the *cas*/*csm* operon.

(d) The numbers indicate the chromosomal coordinates of the *S. epidermidis* RP62a genome that are deleted. The elements that are believed to have mediated the deletion are shown in parenthesis.

(e) Deletion of the adenine in the first position of the *spc1* sequence.

(f) The nucleotide mutation followed by the amino acid mutation are indicated, the numbers indicated nucleotide or amino acid position of the gene or encoded protein, relative to the start codon or initial methionine residue, respectively.

(g) Highlighted and underlined are transconjugants that were chosen for the pairwise competition experiments estimating fitness.

To check for the deletion of the spacer responsible for immunity against pG0400, we amplified the CRISPR array (Figures 2B and 2C). We found 14 transconjugants that contained *spc1* (5/14), *spc1-2* (8/14) or *spc1-2-3* (1/14) deletions (Figure 2B and Table 2), which presumably occurred via recombination of repeat sequences. Given the unique property of CRISPR-Cas systems that enables the acquisition of new spacers [15], [16], [17], [18], [19], this mutation can be considered reversible; i.e. the cell could incorporate a new spacer against the plasmid to eliminate it.

To assay for the presence of inactivating mutations we amplified the full CRISPR-Cas locus. We noted that 24 amplicons contained insertions (Figure 2D). Sequencing of the PCR products identified the presence of the transposable element IS*256* in *csm5* (5/24, 2 unique), *csm6* (15/24, 6 unique) or *cas6* (4/24, 3 unique) (Figure 2B and Table 2), genes that are required for CRISPR immunity [45], [46]. These mutations have a broader effect, since they abolish CRISPR immunity against not only the plasmid but also of all the other targets specified in the repeat-spacer array. We do not know why transposition occurred only into these three genes, since similar insertion sites are present throughout the CRISPR-Cas locus. There are multiple copies of this transposable element in the *S. epidermidis* RP62a genome [34] and its transposition is an important source of genetic diversity in this pathogen [47]. Interestingly, the alternating insertion and excision of IS*256* elements in the *ica* locus of *S. epidermidis* RP62a provides a phase variation mechanism for biofilm production [48], and it is tempting to speculate that a similar mechanism could regulate CRISPR immunity.

Sanger sequencing of the rest of the PCR products revealed mutants with single adenine deletions (14/111) that would abrogate the *spc1* crRNA:target interaction (1/14) or that introduced frameshifts in *cas10* (5/14, same deletion for all), *csm2* (5/14, same deletion for all), *csm4* (1/14), *csm6* (1/14) or *cas6* (1/14) (Figure 2B and Table 2). Four transconjugants contained single-nucleotide substitutions that introduced nonsense mutations in *cas10* (2/4, both unique) or *cas6* (1/4), that together with frameshift mutations will lead to the generation of non-functional truncated Cas proteins. Finally, one mutant contained a G to T transversion in *csm4* that produces a glycine (GGT) to valine (GTT) substitution (escaper R43). Interestingly, this glycine (Gly189) is part of a G-rich loop that is conserved in many *cas* genes belonging RAMP family [49] and our results suggest that this feature is required for CRISPR immunity.

**Figure 3:**
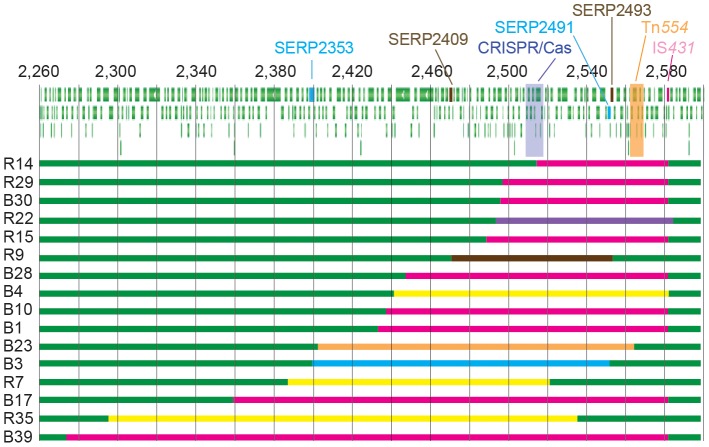
CRISPR escapers accumulate deletions of the CRISPR/Cas region. Schematic representation of the deletions on the *S. epidermidis* RP62a genome. Wild-type sequences are shown in green, deletions mediated by IS*431* in pink, by IS*256* in yellow, by Tn*554* in orange, by recombination between SERP2353 and SERP2491 (96% identical at the nt level) or SERP2409 and SERP2493 (98% identical at the nt level) in light blue or brown, respectively, and by the excision of the SCC*mec* cassette in violet. Numbers represent genomic coordinates in kb.

For 55 of the 111 transconjugants we were unable to amplify any region of the CRISPR-Cas locus and thus we suspected that the full locus was missing. We then designed multiple primers annealing at both flanking regions of the CRISPR-Cas locus and performed PCRs until we obtained amplicons that were sequenced to identify the deleted sequences. In this way we identified 16 different mutants containing deletions ranging from 65,712 (2.5% of the *S. epidermidis* RP62a genome) to 306,488 nt (11.6% of the genome) (Figure 3). Examination of the deletion junctions indicated that these were facilitated either by transposons (IS*431*, 9/16; IS*256*, 3/16; or Tn*554*, 1/16), recombination of homologous regions (SERP2353 and SERP2491, 1/16; SERP2409 and SERP2493, 1/16) or excision of the SCC*mec* gene cassette (adjacent to the CRISPR/Cas locus, 1/16).

### Generality of the “loss of CRISPR” solution

To determine whether conjugation is required for the generation of CRISPR mutants we designed an inducible targeting system. We complemented the Δ*crispr* strain with a plasmid, pLM477, bearing the spacer-repeat region of *S. epidermidis* RP62a under the control of the IPTG-inducible Spac promoter [50]. We conjugated pG0400 into Δ*crispr*/pLM477 cells and plated transconjugants in the presence or absence of IPTG (Figure S1A). As expected, only a few escapers were obtained in plates containing the inducer, but many colonies in its absence (Figure S1B). Most of these colonies were small, suggesting a partial level of interference possibly due to leaky expression of the CRISPR array. One of these small colonies was grown in liquid culture until OD_600_ was 0.2, when IPTG was added. One hour after the addition of the inducer cells were plated in solid media containing IPTG to isolate escapers. We genotyped 30 colonies to find similar results to those observed for the conjugation escapers: a mix of spacer deletions, transposon insertions, point mutations and complete deletions of the CRISPR-Cas locus (Figure S1C and Table S1). The only difference was in the number of spacer deletions, which was higher than the conjugation escapers. We speculate that this may be related to the presence of the CRISPR array on a plasmid as opposed to the chromosome; these deletions are most likely generated by replication slippage and the plasmid replicates more frequently than the chromosome (the estimated copy number of pC194, the parent vector of pLM477, is 15 [51]). These results indicate that the same inactivating CRISPR mutations occur regardless of whether the plasmid is targeted during or after conjugation.

To begin to explore whether the loss of functional CRISPR-Cas loci is unique to the particular *spc1*-*nes* target interaction, we designed a second spacer (*spcA*) that matches a pG0400 gene encoding a hypothetical ORF, but, as opposed to *spc1*, targets the coding strand of this gene (Fig. S2A). To test the efficacy of this new spacer we introduced it into a minimal CRISPR array [45] containing only the leader sequence, followed by the first repeat and *spcA* (pWJ87). We then introduced this plasmid, as well as a similar plasmid containing *spc1* (pWJ28) and the vector control (pC194 [51]), into Δ*crispr* cells and used the transformants as recipients for pG0400 (Figure S2B). *SpcA*-mediated immunity against conjugation was similar to that provided by the control plasmid carrying *spc1*, and similar to the levels obtained using wild-type *S. epidermidis* RP62a as recipient. Twenty escapers were genotyped. First we looked for spacer deletions in the *spcA* plasmid, but found none. Most likely, this is due to the absence of a repeat downstream of the spacer in the complementing plasmids, which would prevent the deletion of a spacer sequence between two repeats by replication slippage. PCR amplification of the CRISPR-Cas locus indicated that 5/20 escapers contained transposon insertions and 12/20 lacked the locus altogether (Fig. S2C). The PCR products derived from the remaining three escapers were sequenced, revealing point mutations in different *cas* genes. The proportion of the different types of mutations is similar to those obtained using the *spc1* target (Figure S2D), thus corroborating the generality of our previous findings.

### Non-functional CRISPR-Cas mutants and deletions abound in *S. epidermidis* RP62a populations

Our results suggest that CRISPR-negative mutants are generated at a high rate. Based on our estimates of Γ (Table 1), the rate at which pG0400 is transferred to the immune host (*S. epidermidis* RP62a) is 10^−4^ to 10^−3^ times the rate at which it is transferred to a permissive strain (*S. epidermidis* Δ*crispr*). In other words, with a probability of 10^−4^ to 10^−3^ CRISPR-Cas positive cells modify the spacer or deactivate or lose the CRISPR-Cas locus. In principle, this can occur either spontaneously or through an unknown process induced by plasmid transfer. To test if these mutants pre-exist in *S. epidermidis* RP62a populations, we performed a fluctuation experiment [52]. We compared the variance of the number of transconjugants obtained in 10 filter matings using 10 different *S. epidermidis* RP62a recipient cultures (each originated from a single colony) with the variance obtained in 10 conjugations using aliquots of a single recipient culture (Table 3). As donors, aliquots of the same *S. aureus*/pG0400 culture were used for all conjugations. If CRISPR mutations are induced during conjugation both variances should be similar. If, on the other hand, CRISPR mutations are pre-existing, the variance for the number of transconjugants observed with 10 independent recipient cultures will be higher than the variance of the 10 controls. This is what we obtained, with the variance/mean ratio for the 10 independent conjugations 50-fold higher than the control value. Even if we exclude the “jackpot”, the conjugation experiment (# 7) that resulted in the higher number of transconjugants, we still obtain a 10-fold higher variance/mean value compared to the control experiment. While we cannot completely rule out that CRISPR mutations are induced during immunity, the results of our fluctuation experiments provide compelling support for the presence of pre-existing CRISPR mutants in *S. epidermidis* RP62a recipients.

**Table 3:** Fluctuation experiment (cfu/ml).

	10 aliquots of the same culture			10 independent cultures		
					
Experiment #(a)	Recipients(b)		Transconjugants	Recipients(b)	Transconjugants	w/o Jackpot(d)
1	2.08×10^8^		147	2.02×10^8^	167	167
2	1.85×10^8^		122	1.87×10^8^	193	193
3	1.68×10^8^		129	1.56×10^8^	80	80
4	1.84×10^8^		187	2.13×10^8^	107	107
5	1.80×10^8^		124	1.49×10^8^	207	207
6	1.90×10^8^		127	1.63×10^8^	213	213
7	1.67×10^8^		158	1.71×10^8^	907(c)	–
8	1.84×10^8^		131	2.01×10^8^	380	380
9	1.58×10^8^		116	1.75×10^8^	127	127
10	1.61×10^8^		93	1.88×10^8^	80	80
Mean			133		246	173
Median			128		180	167
Variance			581		55488	7767
Variance/Mean			4		226	45

(a) All experiments were performed using an aliquot of the same *S. aureus* RN4220/pG0400 donor culture.

(b) The average of two cfu counts of recipients (*S. epidermidis* RP62a) obtained after conjugation is reported. Donors were not enumerated.

(c) Jackpot

(d) Mean, median, variance and variance/mean values were re-calculated omitting the jackpot cfu (907 cfu/ml).

As can be seen in Table 1, the estimated rate parameter of plasmid transfer, Γ, for permissive matings (donors bearing pG0400^mut^ and wild type RP62a recipients, and donors bearing wild type pG0400 plasmid and CRISPR-deletion recipients, Δ*crispr*) are between 3 and 4 orders of magnitude greater than that for donors bearing wild-type plasmids and CRISPR immune RP62a recipients. Since the number of donors is similar for the permissive and non-permissive matings, it seems reasonable to assume that the difference between these matings is the density of the permissive (mutant) recipients. One interpretation of this is that the rate at which mutant recipients with loss-of-function mutations or deletions in the CRISPR-Cas locus, µ per cell per generation, is between 10^−3^ and 10^−4^. Additional support for this interpretation can also be seen from the results of our simulation study with a semi-stochastic model of random mutation for CRISPR-loss and plasmid transfer (Supplemental Text 1).

### Fitness of transconjugants with non-functional or deleted CRISPR-Cas loci

Can the observed inactivations or deletions of the CRISPR-Cas region be a realistic mechanism by which natural populations of immune CRISPR-Cas positive bacteria acquire beneficial plasmids? If they were, we would anticipate that the inactivation or deletion of CRISPR-Cas loci would engender little or no fitness cost on the bacteria and thereby these CRISPR-negative mutants could be maintained in natural populations.

**Figure 4:**
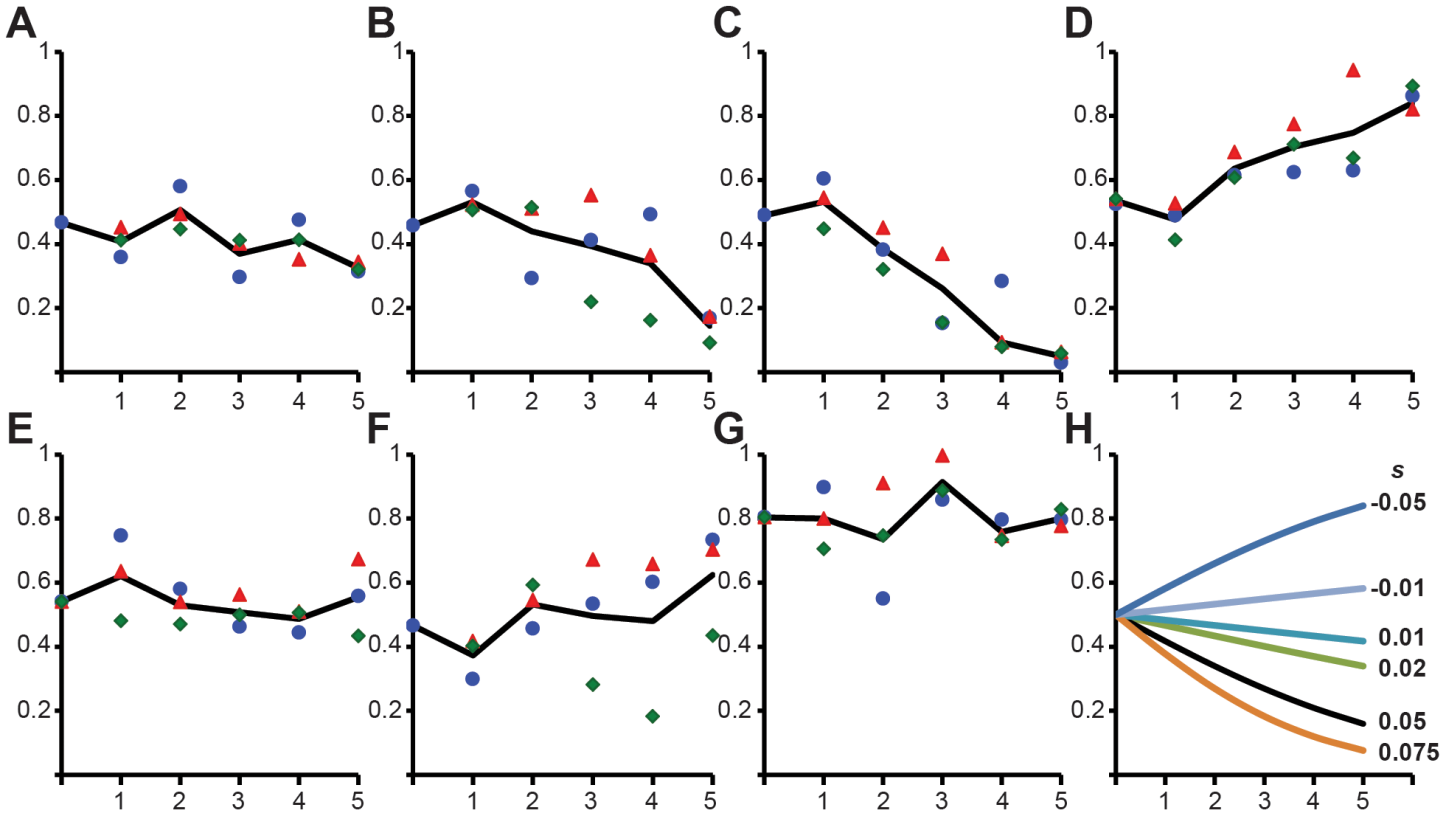
Fitness of CRISPR inactivated and deleted transconjugants. Pair-wise competition between transconjugants carrying different types of CRISPR-Cas mutations or deletions and wild-type *S. epidermidis* RP62a. The change in the relative frequency of plasmid-bearing cells (*y*-axis) is plotted against the number of transfers (one transfer per day, *x*-axis). In all cases the growth of wild-type cells was compared against: (**A**) control wild-type *S. epidermidis* RP62a (pG0400*^mut^*), (**B**) R5, (**C**) R60, (**D**) R14, (**E**) R7, (**F**) B15, (**G**) B39. The black line indicates the average change in relative frequency (the values for each of three independent experiments are shown as a red triangle, blue circle and green rhombus). (**H**) Predicted changes in frequency for different selection coefficients, *s*. These are calculated from the equation, *dq*/*dt*  =  –*q*(1 – *q*)*s*, where *q* is the relative frequency of the plasmid bearing cells and *s* is the selection coefficient (*s*>0 indicates that the plasmid-bearing cells are at a disadvantage and *s*<0 that the plasmid-bearing cells have an advantage). We are assuming 1/100 dilutions or *t* = 6.64 generations in each transfer.

To begin to address this question, we performed pair-wise competition experiments to estimate the fitness of CRISPR-Cas mutants pG0400 transconjugants relative to wild-type *S. epidermidis* RP62a. For these experiments, we selected six transconjugants, R5, R7, R14, R60, B15 and B39, each representing a different type of mutation or deletion found in our study (Table 2). As a control for the fitness effect of the plasmid, we performed a pair-wise competition experiment between *S. epidermidis* RP62a/pG0400*^mut^* and *S. epidermidis* RP62a. The results of these experiments are presented in Figure 4. Also included in this figure are the changes in the frequency of transconjugants with different fitness costs anticipated from population genetic theory (Figure 4H).

The control experiment (Figure 4A) suggests that carriage of the plasmid is relatively neutral. Two transconjugants, R5 and R60, appear to be 5 to 10% less fit than the CRISPR-positive *S. epidermidis* RP62a (Figures 4B and 4C). One transconjugant, R14, is more fit than the wild-type strain (about 5% fitness gain, Figure 4D). The remaining three transconjugants examined, R15, R7 and B39, appeared to be as fit as the competing wild-type strain. These results are particularly striking because mutants R14 and B39 contain deletions that span 66 and 306 kb, representing 2.5 and 11.6%, respectively, of the *S. epidermidis* genome.

### Why we did not see mutant plasmids escaping CRISPR immunity

As noted in [25] and used here as a control, a plasmid engineered to contain mutations in the *nes* target region is capable of transferring to otherwise immune *S. epidermidis* RP62a recipients with high efficiency. Why then did we not detect CRISPR-escape mutant (CEM) plasmid in our “offer they can’t refuse” plasmid transfer experiments? We postulate that the reason for this is simply one of rates. The rate at which these plasmid mutations are generated is vastly lower than the combined rate of loss of CRISPR immunity by spontaneous mutation (including the insertion of transposable elements) and deletion. To illustrate this we use computer simulations of a semi-stochastic model of the population dynamics of conjugative plasmids in a CRISPR-positive population immune to the carriage of that plasmid (described in Supplementary Text 2).

In these simulations there are six distinct populations of bacteria: recipients that are immune to the receipt of the plasmid, CP, and those that are not because of spacer mutations and/or the loss or deletion of a functional CRISPR-Cas locus, CN; donors bearing the wild-type plasmid targeted by the CRISPR-Cas system, D1, or a mutated plasmid that can escape CRISPR immunity, D2; and transconjugants T1 that are produced by matings between D1 and CN, that can transfer plasmid to CN but not CP recipients, and T2 produced by matings between D2 or T2 and either CP or CN recipients. Wild-type CP cells lose CRISPR-Cas mediated immunity with a probability µ per cell per hour, CP→CN. CEM plasmids are generated with a probability ν per cell per hour, D1→D2.

**Figure 5:**
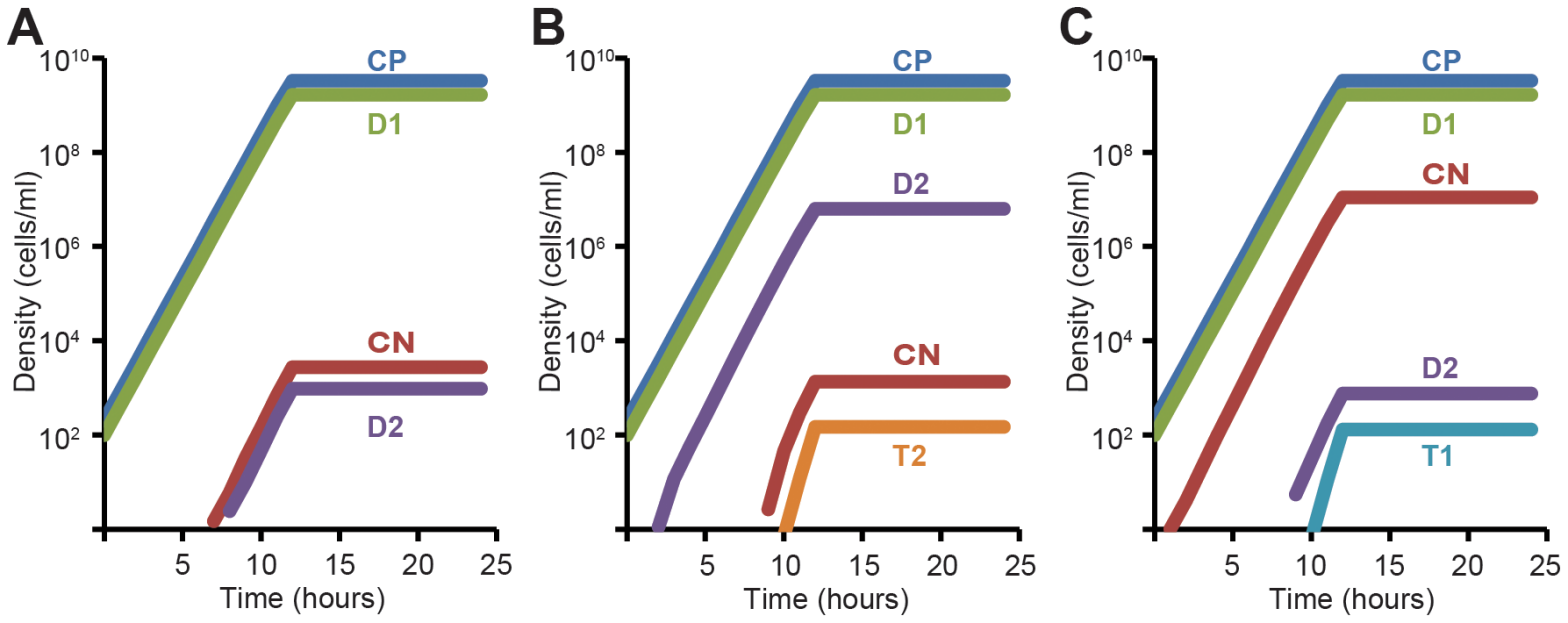
Simulation of plasmid competition with CRISPR-mediated immunity. Changes in the densities of different populations over time are plotted. Standard parameters (defined in Supplementary Text 2) are: ν = 1.4, *e* = 5×10^−7^, *k* = 1, γ = 10^−14^, initial values, R = 2500, CP = 200, D1 = 100, CN = D1 = D2 = T1 = T2 = 0. (A) Same rate of CRISPR loss (µ) and plasmid escape mutations (ν), µ = ν = 10^−7^. (B) High rate of plasmid escape mutants, µ = 10^−7^, ν = 3×10^−4^. (C) High rate of CRISPR loss or deletion mutations, µ = 3×10^−4^, ν = 10^−7^.

Using population densities, growth and plasmid transfer rate parameters in the range estimated for matings between permissive donors and recipients, we considered three different scenarios: one where both mutation rates are low and equal µ = ν = 10^−7^ (Figure 5A), one where the rate of generation of CEM plasmids considerable exceeds the rate of CRISPR loss, µ = 10^−7^ and ν = 3×10^−4^ (Figure 5B), and one where the rate of generation of CRISPR-Cas mutants considerably exceeds the rate of CEM mutations in the plasmid, µ = 3×10^−4^ and ν = 10^−7^ (Figure 5C). Twenty independent runs were made with each set of parameters. In the first scenario the density of both T1 and T2 transconjugants remained less than 1 after 24 hours (Figure 5A). In the second, D2 donors are produced at a high rate and as a result there are substantial numbers of T2, CEM plasmid transconjugants, at 24 hours (99.5±27.0 cfu/ml) (Figure 5B). Although CRISPR mutants, CN, are produced, their densities remain too low to be converted into T1 transconjugants. Finally, in the third scenario at 24 hours there are a substantial number of transconjugants with the wild type plasmid on CRISPR-negative mutants, T1 (102.3±27.5 cfu/ml) (Figure 5C). This third scenario is the most consistent with our experimental and other simulation results. From our plasmid transfer data (Table 1) and fluctuation test simulation (Supplemental Text 1), we calculate µ to be between 10^−4^ and 10^−3^ per cell per hour (see above). Based on what we would anticipate for nucleotide substitution rates in bacteria [53], [54], [55], a ν = 10^−7^ per plasmid per generation would be a rather high approximation for a single base substitution mutation rate in the 35 base pair target region of the *nes* gene of pGO400. Thus our last simulation explains how these differences in rates of generation of CRISPR vs. plasmid mutants prevented us to obtain transconjugants carrying pG0400 with mutated targets.

## Discussion

The widespread presence of anti-plasmid spacers in bacteria is a puzzling aspect of CRISPR-Cas adaptive immunity. Plasmids can carry antibiotic resistance or other genes that can be beneficial and under some conditions essential to the survival of bacteria. CRISPR-Cas systems that target these elements markedly reduce the ability of these organisms to acquire and maintain these accessory genetic elements and thereby adapt to and survive in their environment. How do bacteria deal with this downside of CRISPR immunity? Our experiments with *S. aureus*, *S. epidermidis* and their plasmid pG0400 predict that the most likely mechanism is by mutations in the CRISPR-Cas locus that modify or delete the spacer responsible for the immunity to the plasmid or that abrogate the function or result in the deletion of the locus. Inactivation or loss of CRISPR-Cas loci has been observed in several organisms [56], [57], [58]; our study reveals the molecular mechanisms responsible for this loss. Our results also suggest that, at least in *S. epidermidis*, mutations that eliminate CRISPR immunity are spontaneous and occur at substantial rates (between 10^−4^ and 10^−3^ per cell per generation) and are unlikely to be induced by CRISPR immunity during conjugation or after receipt of the plasmid; i.e. CRISPR-deficient mutants exist in the population.

We believe that these results also provide an explanation for why, in marked contrast to the results of analogous experiments with phage [7], [8], [9], [39], [40], the evasion of CRISPR immunity is not by mutations in the target region of the infecting plasmid but by mutations on the CRISPR-Cas locus itself. As demonstrated in [25], target mutations allow pG0400 to become established and maintained in immune strains of *S. epidermidis* RP62a. We postulate, and support with computer simulations using our estimated parameters, that these plasmid mutations were not observed in our experiments and by extension in natural populations because they occur at vastly lower rates than host mutations that eliminate CRISPR immunity or the CRISPR loci at large. At this juncture, we do not know the mechanisms responsible for the high rate of CRISPR inactivation, however it is in accordance with previous results for loss of function mutations in the *ica* locus (responsible for biofilm formation) of *S. epidermidis* RP62a, located in the same region of the genome of this bacterium (170 kb downstream of the CRISPR-Cas locus, both loci were deleted in R35 and B39): Ziebuhr and coworkers reported a 10^−5^ frequency for the loss of the biofilm phenotype [47], [48]. If this higher rate of CRISPR inactivating mutations is a general property of CRISPR loci and not exclusive to *S. epidermidis*, then the evolutionary race between CRISPR-Cas loci and their targets will be driven by mutations in the invader in the case of lytic phages, but by mutations in the host in the case of beneficial plasmids or mobile genetic elements.

Albeit not evidence that in natural settings non-functional mutants and deletions of the CRISPR-Cas region can have little or no fitness cost, the results of our pair-wise competition experiments suggest that this may be the case. Although some of the CRISPR-deficient transconjugants tested were less fit than wild-type, others were either as fit or even more fit. Particularly striking were transconjugants R14, which in spite of harboring a deletion of 2.5% of the *S. epidermidis* RP62a genome displayed a fitness gain compared to wild-type, and B39, which despite a deletion of 11.6% of its genome we were unable to detect a fitness cost. On first consideration, it may seem unlikely that the genomes of bacteria and archaea contain whole regions that can be readily lost or deactivated without imposing a substantial cost on the rate of survival, replication or competitive performance [59]. It does, however, seem more plausible if those labile regions bore few if any genes that are required for the survival and replication of the cell (so called “house-keeping genes”) but rather served as a home of genes and genetic elements that are not universally essential, i.e. useful or even essential for specific situations. The latter will be the case for many of the genes that are acquired by horizontal gene transfer and there is evidence supporting the idea that the region surrounding the CRISPR-Cas locus of *S. epidermidis* RP62a is a hotspot for the incorporation of laterally transferred genes. In addition to the CRISPR-Cas system, this region contains other genes and genetic elements that are likely to have been transferred from other staphylococci [31], [34]. Included among them are the arginine catabolic mobile element (ACME), the *ica* operon required for biofilm formation, various antimicrobial resistance genes (erythromycin, fosfomycin, arsenic, penicillin and streptomycin), a type I restriction-modification system, a composite transposon (Tn*554*), and a type II staphylococcal chromosomal cassette *mec* (SCC*mec* II). Due to their “foreign” nature, perhaps these genes are only essential for survival in particular conditions, different than laboratory conditions. Deletion of CRISPR-Cas loci has been observed in different archaeal species [56], [57] and, although these studies did not determine the extension of the deletions nor their fitness cost, it is conceivable that adjacent regions are lost as well. If so, the concurrent elimination of CRISPR-Cas loci and its surrounding genes upon the transfer of beneficial mobile genetic elements targeted by the system may be a general phenomenon.

We interpret the results of these ‘offer they can’t refuse’, along with other experiments and our simulations, as support for the broader hypothesis that the CRISPR-Cas region is labile. Bacteria and archaea can deactivate and/or lose and reactivate and/or acquire CRISPR-Cas loci with little or no cost in their intrinsic fitness. As a result of this opposing positive (phage-dependent) and negative (dependent on plasmids and accessory genetic elements that augment fitness) selective forces the CRISPR-Cas loci of bacteria and archaea are in a continuous state of flux. Taken at large, the theoretical and experimental results of this study and the postulated flux suggest an explanation for the profound variation in number, function and existence of CRISPR-Cas loci within and between species of bacteria and archaea [60], and why pathogenic strains of enterococci [58], and perhaps other pathogens that rely on the horizontal transfer of virulence and/or resistance genes are likely to lack functional CRISPR-Cas loci.

Fluctuations in spacer content of CRISPR arrays of bacteria and archaea have been observed in natural populations [22], [61]. Presumably, the removal of spacer-repeat units occurs through recombination of repeat sequences. As demonstrated here, modified and lost spacers can be strongly favored when these spacers target beneficial DNA. Changes in spacer content alone, however, does not inactivate CRISPR immunity, since the system is able to constantly acquire new spacers [8], [15], [16], [17], [18], [19]. A second, more dramatic level of flux is the complete inactivation/loss and reactivation/gain of CRISPR-Cas systems. Prokaryotes acquire CRISPR-Cas loci by horizontal gene transfer [40], [41], [42], [62]. These loci ascend and become established in specific lineages as a consequence of their encounters with phage (the upside of CRISPR immunity). However they can be lost or become non-functional in environments where the carriage plasmids and other horizontally transmitted genes and genetic elements provide a selective advantage (the downside of CRISPR). Another possibility for the loss of CRISPR function would be the acquisition of self-targeting spacers, previously suggested by Sorek and colleagues [24]. In both cases the spacers responsible for immunity to plasmids and other host genes are picked up and become established primarily accidently, perhaps by transducing bacteriophages [17], and may be maintained in the population through genetic linkage to an essential anti-phage spacer.

In conclusion, we postulate that bacteria and archaea adapt to the downside of CRISPR immunity by selecting for the modification of the targeting spacers responsible for the immunity or the deactivation or deletion of the CRISPR-Cas loci. This would happen in situations where CRISPR immunity blocks the transfer of exogenous genes and genetic elements that are needed for the survival and adaptation of the host to its environment. If true, this hypothesis predicts that organisms targeting potentially beneficial mobile genetic elements will accumulate mutations in their CRISPR-Cas loci. To begin to test this hypothesis, we searched for organisms containing spacers that match endogenous plasmids or prophages [24] and assessed the integrity of their CRISPR loci. We included prophages as they represent another important source of beneficial genes for bacteria [63], [64]. In this preliminary search we found three examples that corroborate the predictions of our results (Figure S3). *Escherichia coli* UTI89 [65] carries an endogenous plasmid, pUTI89, and a type I-F CRISPR-Cas locus with a spacer matching this plasmid and a nonsense mutation in *csy2* (Figure S3A), similar to the case of escapers R52, R66 and B37 in our findings. Another gram-negative bacterium, *Dichelobacter nodosus* VCS1703A [66], hosts a Mu-like prophage that is targeted by a type I-F CRISPR system missing the *cas1* and *cas3* genes (Figure S3B). Finally, the gram-positive organism *Lactobacillus brevis* ATCC 367 [67] harbors a prophage matched by a spacer in an orphan CRISPR array, i.e. *cas* genes cannot be found in the genome of this bacterium (Figure S3C). These are some of the most evident cases of CRISPR inactivation, but most likely there are many more. For example, many *cas* genes containing inactivating point mutations similar to the *csm4* mutation in escaper R43, are at the moment difficult to be identified as inactive alleles by bioinformatics analysis. In addition, full deletions of CRISPR-Cas systems normally occur without leaving evidence of their past presence in a genome. Our predictions should also be testable experimentally by subjecting populations of CRISPR-Cas bacteria that are immune to both plasmids and phages to sequential episodes where they are confronted with a plasmid that increase fitness and a lethal phage. As information about the structure and function of CRISPR-Cas loci increases, as it certainly will, we predict that the signatures of these fluxes in the function, acquisition by HGT and deletion of the CRISPR-Cas loci, will become increasingly evident.

## Materials and Methods

### Bacterial strains and growth conditions

*S. epidermidis* RP62a [34] and *S. aureus* RN4220/pG0400 [68] were grown in brain heart infusion (BHI) media (Difco) at 37°C. When required, the medium was supplemented with neomycin (15 µg/ml) for selection of *S. epidermidis* RP62a, mupirocin (5 µg/ml) for selection of *S. aureus* RN4220/pGO400, or both, for the selection of *S. epidermidis* RP62a/pG0400 transconjugants. Chloramphenicol (10 µg/ml) was used for the selection of plasmids pC194, pLM477, pWJ28 and pWJ87. IPTG (isopropyl β-D-1-thiogalactopyranoside) was used at a final concentration of 1 mM for induction of crRNA transcription in cells harboring pLM477.

### Plasmid construction

Plasmid pLM477 was constructed by the cloning of a promoter-less *S. epidermidis* RP62a CRISPR array into pLM9. The insert was amplified using primers AM1 and L299, cut with SalI and NheI and ligated with pLM9 cut with XhoI (compatible ends with SalI) and NheI. pLM9 was generated by ligating the KpnI/HindIII restriction fragment of pMutinHA [50], containing the Pspac promoter and the *lacI* repressor, with a PCR product obtained using primers L270 and L271 and the staphylococcal plasmid pC194 [51] as template, cut with the same restriction enzymes. Plasmids pWJ28 and pWJ87 were constructed by PCR amplification of pCRISPR(wt) [69] with primer pairs A10/L55 and W591/W592, respectively, followed by phosphorylation of the 5′ ends with T4 Polynucleotide Kinase (New England Biolabs), and circularization using T4 DNA ligase (New England Biolabs). In all cases, ligation products were transformed into *S. aureus* OS2 [70]. Plasmids were isolated, their sequence corroborated by Sanger sequencing, and transformed into *S. epidermidis* Δ*crispr* as described previously [25]. Table S3 contains all the primers used in this study.

### Conjugation

Conjugation was carried out by filter mating as described previously [25], but using more donors and recipients. Briefly, donor (*S. aureus* RN4220/pG0400) and recipient (*S. epidermidis* RP62a) cells were cultured in BHI medium with necessary antibiotics at 37°C overnight. 10^9^ donors and 5×10^8^ recipients were mixed in 5ml of fresh BHI medium and vacuum-filtered through 0.45 µM filters (Millipore). Filters were incubated on BHI agar plates at 37°C for 18 hours and bacteria were resuspended in 3 ml of fresh BHI. Serial dilutions were then plated on BHI agar containing the appropriate antibiotics for the enumeration of donors, recipients or transconjugants (Table 1).

### Genotyping

Transconjugants DNA was extracted and used as template for PCRs with primers L50/L6 for amplification of the CRISPR array, L19/L340 and L23/L106 for amplification of upstream or downstream halves of the CRISPR-cas locus, respectively, and L70/L71 for amplification of the pG0400 *nickase* gene (*spc1* target). All escapers contained the expected pG0400 PCR product. Many escapers showed CRISPR locus PCR products with non-wild-type sizes. These were sequenced to corroborate spacer deletions and transposon insertions. CRISPR locus PCR products were not detected with many other escapers and were suspected of containing deletions encompassing the locus. These were analyzed with a set of primers distributed along the CRISPR-cas region of the *S. epidermidis* RP62a chromosome. The exact deleted sequence was determined by sequencing of PCR products that were obtained by amplifying the deletion junction. Transconjugants without indications of deletions or insertions were first checked for the presence of an intact nickase target by sequencing of the L70/L71 PCR product. As none showed any target mutations, L19/L340 and L23/L106 PCR products were sequenced to look for mutations in the CRISPR-Cas locus. Table S3 contains all the primers used in this study.

### Fluctuation experiment

Ten independent conjugation experiments were carried out by using *S. epidermidis* RP62a recipient cultures starting from a single colony or 10 different colonies. In all cases a single *S. aureus* RN4220/pG0400 donor culture was used. Conjugation experiments were performed as described earlier with the modification that erythromycin (10 µg/ml) was used in addition to neomycin to select for *S. epidermidis* RP62a transconjugants and eliminate the few neomycin- and mupirocin-resistant *S. aureus* donors that were detected in our first conjugation assays (R21 and R26, see Table 2).

### Pairwise competition experiments

Overnight cultures of wild-type *S. epidermidis* RP62a and the competing transconjugant strain were mixed, usually at a ratio of 1∶1. The cultures were grown at 37°C with shaking for 24 hours, vigorously vortexed and 100 µl aliquots transferred to fresh flasks containing 10 ml of fresh BHI media. This serial transfer process was repeated daily for 5 transfers. The total densities of cells in these cultures and the densities of plasmid-bearing cells were estimated at each transfer by serial dilutions and plating on BHI and BHI containing mupirocin (BHI-mup) agar respectively. The relative frequency of plasmid-bearing cells was calculated from the ratio of CFU (colony forming units) estimates of the densities on BHI-mup and BHI.

## Supporting Information

Figure S1 Characterization of escapers generated during CRISPR immunity against a resident pG0400 plasmid. (A) Conjugative transfer of pG0400 into *S. epidermidis* Δ*crispr*/pLM477. Colony forming units (cfu) for recipients and transconjugants are indicated. The average cfu count of three independent conjugation assays is indicated; error bars indicate one standard deviation. Conjugation efficiency (Conj. Eff.) is calculated as the transconjugants/recipients ratio. pLM477 is a chloramphenicol-resistant plasmid that harbors the CRISPR array deleted in the host under the control of an IPTG-inducible promoter. Conjugations were carried as described for experiments using wild-type *S. epidermidis* RP62a as recipient, but recipients and transconjugants were plated on solid media with or without IPTG for their enumeration. The inducer activates CRISPR immunity and only “escaper” transconjugants are recovered. In the absence of IPTG, the lack of CRISPR immunity allows the recovery of colonies containing both an intact CRISPR-Cas system and the targeted plasmid. (B) One of such colonies was inoculated in liquid media and IPTG was added during the beginning of the exponential growth to trigger CRISPR immunity against the resident pG0400 plasmid. Addition of IPTG results in the expression of a small crRNA antisense to the pG0400 *nickase* (*nes*) target (both in pink). (C) One hour after induction of CRISPR immunity bacteria were plated on solid media containing mupirocin and chloramphenicol to select for pG0400 and pLM477, respectively, as well as 1 mM IPTG. 30 colonies were genotyped (not shown) to determine the presence of pG0400 and/or CRISPR-Cas mutations. The different mutations found and their proportions are shown. Detailed genotypes of these colonies are described in Supplementary Table S1.
(TIF)

Figure S2 Characterization of escapers generated during CRISPR immunity against a second target in pG0400. (A) Different locations of *spc1* and *spcA* targets on the pG0400 genome. *Spc1* crRNA matches a region in the template strand of the *nickase* (*nes*) gene of this plasmid. *SpcA* crRNA matches a region in the coding strand of a gene encoding a hypothetical ORF separated by 3.5 kb from the *spc1* target. DNA sequences are highlighted in grey. (B) Conjugative transfer of pG0400 into *S. epidermidis* Δ*crispr* recipients carrying either pWJ28 (expressing *spc1* crRNA), pWJ87 (expressing *spcA* crRNA) or pC194 (the empty vector control). Colony forming units (cfu) for recipients and transconjugants are indicated. (C) 20 *S. epidermidis* Δ*crispr*/pWJ87/pG0400 transconjugants colonies that evaded *SpcA*-mediated CRISPR immunity were genotyped to determine the presence of pG0400 and/or pWJ87 or CRISPR-Cas mutations. The different mutations found and their proportions are shown. Detailed genotypes of these colonies are described in Supplementary Table S2. (D) PCR analysis of the *cas* gene region of escapers using primers L23/L106. DNA from transconjugants WJe101 to 120 was used as template. M, DNA marker; Δ, amplification using *S. epidermidis* Δ*crispr* template DNA. IS*256* transposon insertions are detected as larger PCR products (lanes 104, 105, 110, 111, 116). Deletions of the CRISPR-Cas locus are detected as a lack of PCR product. The CRISPR-Cas locus from transconjugants that did not display a change in PCR product size was subject to Sanger sequencing to detect mutations.
(TIF)

Figure S3 Examples of CRISPR inactivation in available genomes. (A) *E. coli* strain UTI89 harbors a CRISPR-Cas locus containing a spacer that matches a region in the resident conjugative plasmid pUTI89. The sequence as well as chromosomal and plasmid coordinates of the spacer and target, respectively, are shown. The *csy2* gene contains a premature stop codon (TAG) that would inactivate CRISPR immunity. Other strains, namely ED1a, O83:H1 str. NRG 857C and LF82, contain a wild-type copy of the gene with a CAG (glutamine) codon in the same position. (B) *Dichelobacter nodosus* VCS1703A contains a CRISPR-Cas system that targets a resident Mu-like prophage; the sequence and genomic coordinates of spacer and target are shown. However, this system is missing the *cas1* and *cas3* genes commonly present in other similar CRISPR loci (belonging to the subtype I-F group). These are replaced by the *fba* gene, encoding for fructose-biphosphate aldolase. (C) In the case of *Lactobacillus brevis* ATCC 367 an orphan CRISPR array targets a resident prophage; the sequence and chromosomal coordinates for the spacer and target are shown. The spacer-repeat array is flanked by genes *lvis0915* and *lvis0916* (upstream) and *upf0150* and *lytR* (downstream), and there are no *cas* genes elsewhere in this strain.
(TIF)

Table S1 Genotype of cells that escape induction of CRISPR immunity against a resident pG0400 plasmid.
(DOCX)

Table S2 Genotype of cells that escape *spcA*-mediated CRISPR immunity.
(DOCX)

Table S3 Primers used in this study.
(DOCX)

Text S1 A Model for random generation of CRISPR-negative recipients and plasmid transfer.
(DOCX)

Text S2 A model for the population dynamics of conjugative plasmids in an immune CRISPR-positive population.
(DOCX)
