# Supplementary figures and images for: Dealing with the Evolutionary Downside of CRISPR Immunity: Bacteria and Beneficial Plasmids
Source: PLoS Genet. 2013 Sep 26;9(9):e1003844. doi: 10.1371/journal.pgen.1003844 (PMC3784566; doi:10.1371/journal.pgen.1003844)

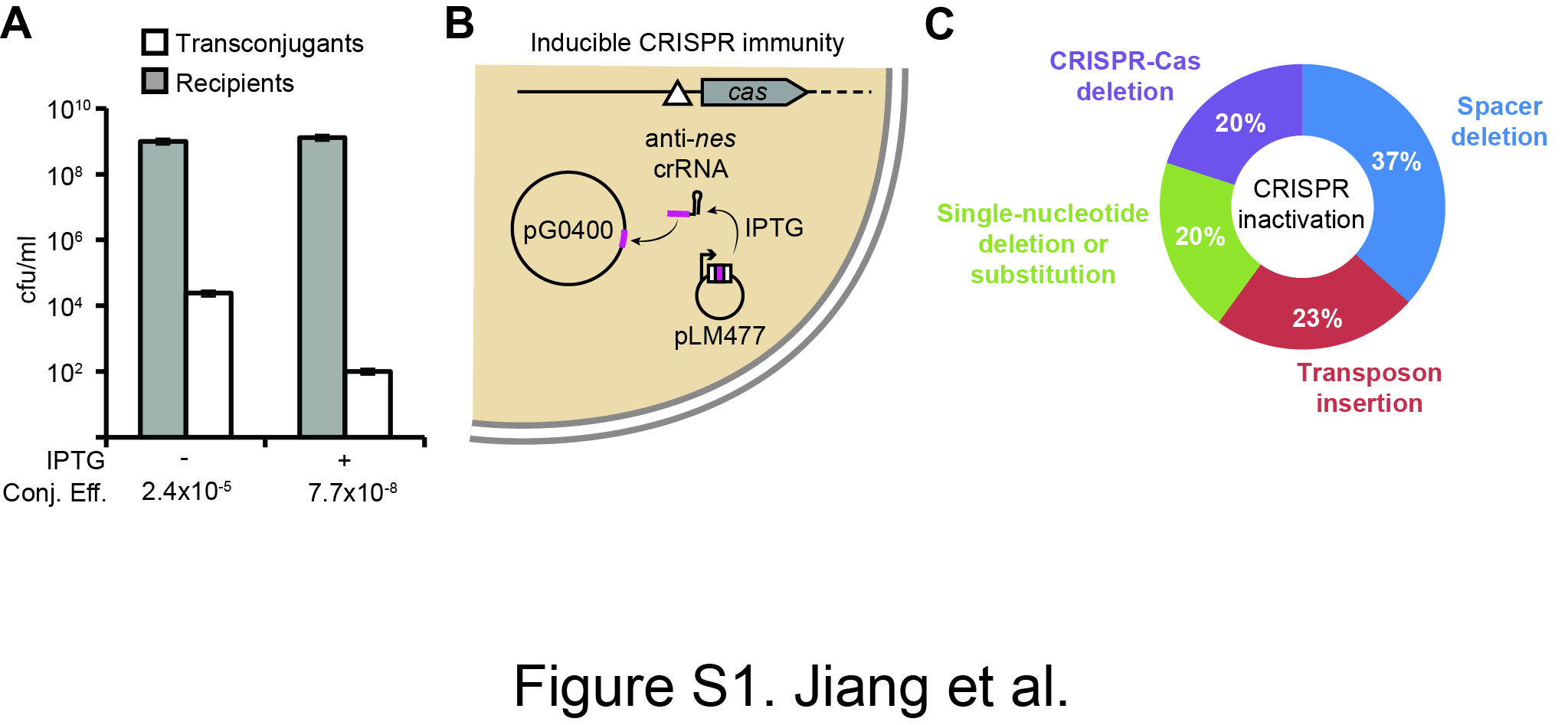

Supplement: Figure S1 — Characterization of escapers generated during CRISPR immunity against a resident pG0400 plasmid. (A) Conjugative transfer of pG0400 into S. epidermidis Δcrispr/pLM477. Colony forming units (cfu) for recipients and transconjugants are indicated. The average cfu count of three independent conjugation assays is indicated; error bars indicate one standard deviation. Conjugation efficiency (Conj. Eff.) is calculated as the transconjugants/recipients ratio. pLM477 is a chloramphenicol-resistant plasmid that harbors the CRISPR array deleted in the host under the control of an IPTG-inducible promoter. Conjugations were carried as described for experiments using wild-type S. epidermidis RP62a as recipient, but recipients and transconjugants were plated on solid media with or without IPTG for their enumeration. The inducer activates CRISPR immunity and only “escaper” transconjugants are recovered. In the absence of IPTG, the lack of CRISPR immunity allows the recovery of colonies containing both an intact CRISPR-Cas system and the targeted plasmid. (B) One of such colonies was inoculated in liquid media and IPTG was added during the beginning of the exponential growth to trigger CRISPR immunity against the resident pG0400 plasmid. Addition of IPTG results in the expression of a small crRNA antisense to the pG0400 nickase (nes) target (both in pink). (C) One hour after induction of CRISPR immunity bacteria were plated on solid media containing mupirocin and chloramphenicol to select for pG0400 and pLM477, respectively, as well as 1 mM IPTG. 30 colonies were genotyped (not shown) to determine the presence of pG0400 and/or CRISPR-Cas mutations. The different mutations found and their proportions are shown. Detailed genotypes of these colonies are described in Supplementary Table S1. (TIF) [file pgen.1003844.s001.tif]

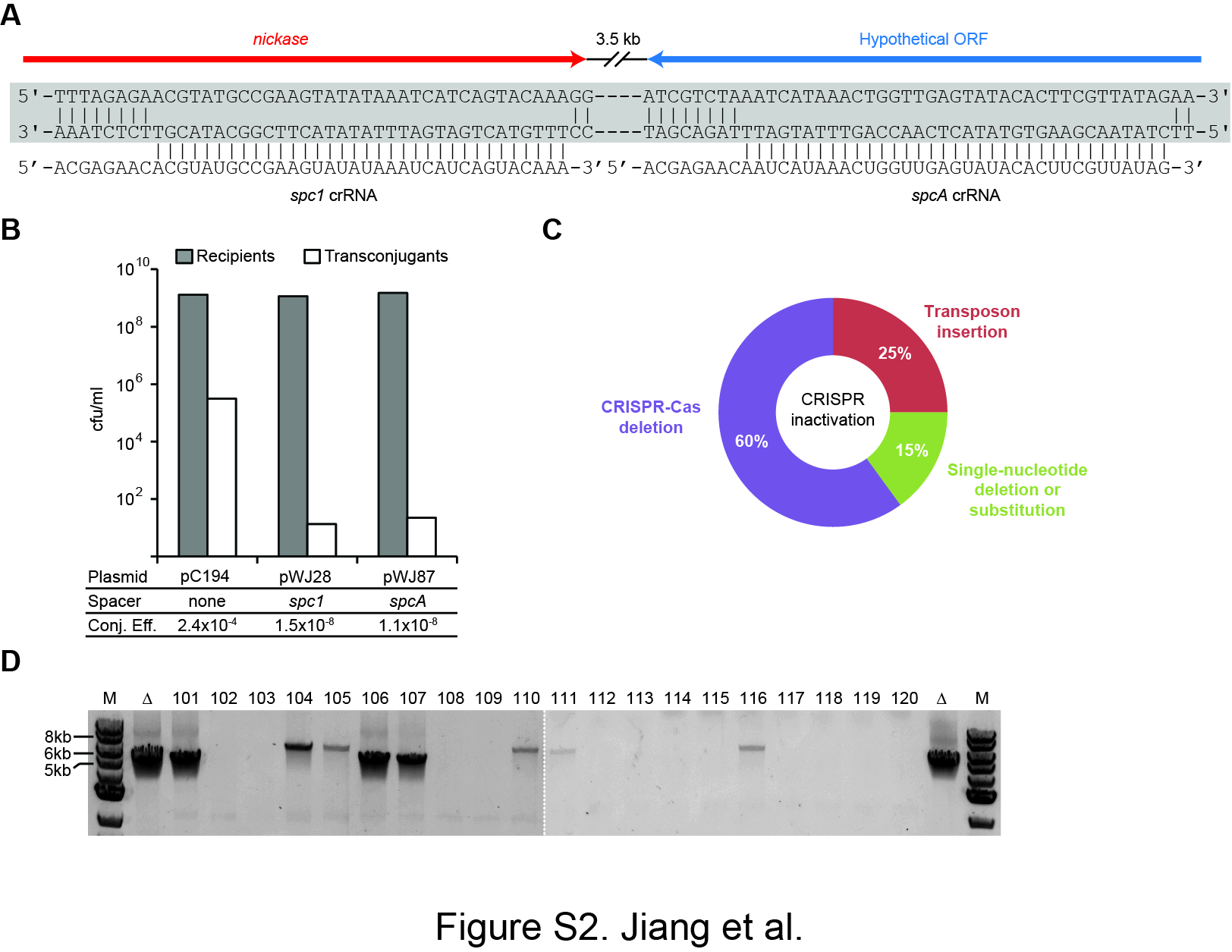

Supplement: Figure S2 — Characterization of escapers generated during CRISPR immunity against a second target in pG0400. (A) Different locations of spc1 and spcA targets on the pG0400 genome. Spc1 crRNA matches a region in the template strand of the nickase (nes) gene of this plasmid. SpcA crRNA matches a region in the coding strand of a gene encoding a hypothetical ORF separated by 3.5 kb from the spc1 target. DNA sequences are highlighted in grey. (B) Conjugative transfer of pG0400 into S. epidermidis Δcrispr recipients carrying either pWJ28 (expressing spc1 crRNA), pWJ87 (expressing spcA crRNA) or pC194 (the empty vector control). Colony forming units (cfu) for recipients and transconjugants are indicated. (C) 20 S. epidermidis Δcrispr/pWJ87/pG0400 transconjugants colonies that evaded SpcA-mediated CRISPR immunity were genotyped to determine the presence of pG0400 and/or pWJ87 or CRISPR-Cas mutations. The different mutations found and their proportions are shown. Detailed genotypes of these colonies are described in Supplementary Table S2. (D) PCR analysis of the cas gene region of escapers using primers L23/L106. DNA from transconjugants WJe101 to 120 was used as template. M, DNA marker; Δ, amplification using S. epidermidis Δcrispr template DNA. IS256 transposon insertions are detected as larger PCR products (lanes 104, 105, 110, 111, 116). Deletions of the CRISPR-Cas locus are detected as a lack of PCR product. The CRISPR-Cas locus from transconjugants that did not display a change in PCR product size was subject to Sanger sequencing to detect mutations. (TIF) [file pgen.1003844.s002.tif]

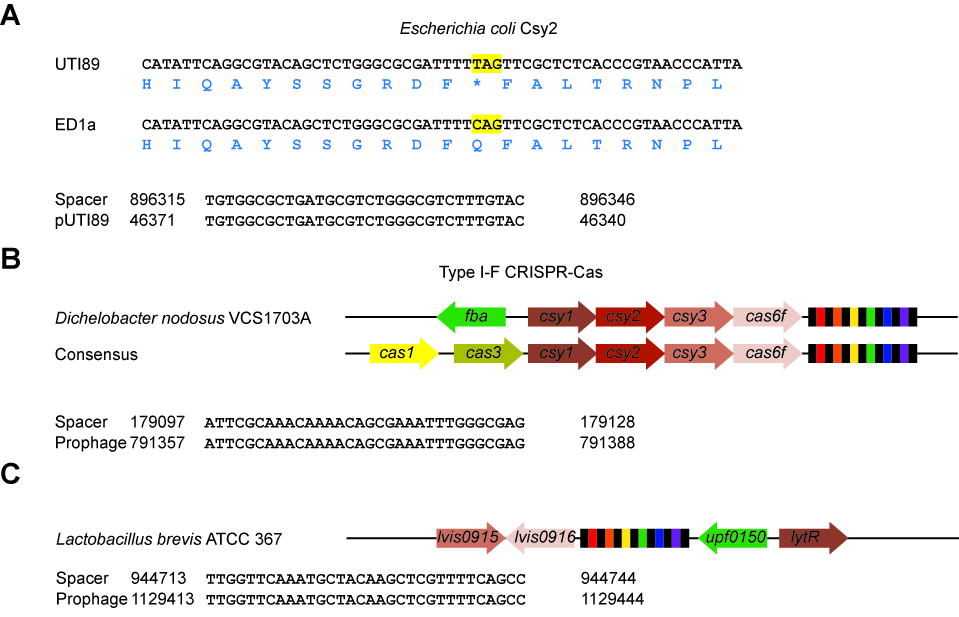

Supplement: Figure S3 — Examples of CRISPR inactivation in available genomes. (A) E. coli strain UTI89 harbors a CRISPR-Cas locus containing a spacer that matches a region in the resident conjugative plasmid pUTI89. The sequence as well as chromosomal and plasmid coordinates of the spacer and target, respectively, are shown. The csy2 gene contains a premature stop codon (TAG) that would inactivate CRISPR immunity. Other strains, namely ED1a, O83:H1 str. NRG 857C and LF82, contain a wild-type copy of the gene with a CAG (glutamine) codon in the same position. (B) Dichelobacter nodosus VCS1703A contains a CRISPR-Cas system that targets a resident Mu-like prophage; the sequence and genomic coordinates of spacer and target are shown. However, this system is missing the cas1 and cas3 genes commonly present in other similar CRISPR loci (belonging to the subtype I-F group). These are replaced by the fba gene, encoding for fructose-biphosphate aldolase. (C) In the case of Lactobacillus brevis ATCC 367 an orphan CRISPR array targets a resident prophage; the sequence and chromosomal coordinates for the spacer and target are shown. The spacer-repeat array is flanked by genes lvis0915 and lvis0916 (upstream) and upf0150 and lytR (downstream), and there are no cas genes elsewhere in this strain. (TIF) [file pgen.1003844.s003.tif]
