# Supplementary material for: Dealing with the Evolutionary Downside of CRISPR Immunity: Bacteria and Beneficial Plasmids
Source: PLoS Genet. 2013 Sep 26;9(9):e1003844. doi: 10.1371/journal.pgen.1003844 (PMC3784566; doi:10.1371/journal.pgen.1003844)
Supplement: Table S1 — Genotype of cells that escape induction of CRISPR immunity against a resident pG0400 plasmid. (DOCX) [file pgen.1003844.s004.docx]

**Table S1. Genotype of cells that escape induction of CRISPR immunity against a resident pG0400 plasmid.**

| **Transconjugant** | **Genotype** |
| --- | --- |
| WJ1, 3, 4, 5, 6, 2, 6, 19, 20, 24, 28 | Δ*spc1* |
| WJ2, 7, 10, 13, 17, 27, 30 | IS*256* insertion^(a)^ |
| WJ8, 9 | *csm5*(C809T; T270I)^(b)^ |
| WJ11, 14 | *csm5*(G937A; G313R)^(b)^ |
| WJ15, 21, 23, 25, 26, 29 | ΔCRISPR-Cas^(c)^ |
| WJ18 | *csm3*(C437A; S146Y)^(b)^ |
| WJ22 | *cas10*(A1731G, Q577)^(b,d)^ |

1. Transposon insertion was determined as a PCR product of a size 1 kb greater than expected.
2. The nucleotide mutation followed by the amino acid mutation are indicated, the numbers indicated nucleotide or amino acid position of the gene or encoded protein, relative to the start codon or initial methionine residue, respectively.
3. Deletion of the entire CRISPR-Cas locus was determined as the lack of a PCR product for this region.
4. Synonymous mutation.
