# Supplementary material for: Dealing with the Evolutionary Downside of CRISPR Immunity: Bacteria and Beneficial Plasmids
Source: PLoS Genet. 2013 Sep 26;9(9):e1003844. doi: 10.1371/journal.pgen.1003844 (PMC3784566; doi:10.1371/journal.pgen.1003844)
Supplement: Table S2 — Genotype of cells that escape spcA-mediated CRISPR immunity. (DOCX) [file pgen.1003844.s005.docx]

**Table S2. Genotype of cells that escape *spcA*-mediated CRISPR immunity.**

| **Transconjugant** | **Genotype** |
| --- | --- |
| WJe101 | *csm4*(G697T; E233Stop)^(a)^ |
| WJe102, 103, 108, 109, 112, 113, 114, 115, 117, 118, 119, 120 | ΔCRISPR-Cas^(b)^ |
| WJe104, 105, 110, 111, 116 | IS*256* insertion^(c)^ |
| WJe106 | *cas10*(G566A, W189Stop)^(a)^ |

1. The nucleotide mutation followed by the amino acid mutation are indicated, the numbers indicated nucleotide or amino acid position of the gene or encoded protein, relative to the start codon or initial methionine residue, respectively.
2. Deletion of the entire CRISPR-Cas locus was determined as the lack of a PCR product for this region.
3. Transposon insertion was determined as a PCR product of a size 1 kb greater than expected.
