# Supplementary material for: Dealing with the Evolutionary Downside of CRISPR Immunity: Bacteria and Beneficial Plasmids
Source: PLoS Genet. 2013 Sep 26;9(9):e1003844. doi: 10.1371/journal.pgen.1003844 (PMC3784566; doi:10.1371/journal.pgen.1003844)
Supplement: Table S3 — Primers used in this study. (DOCX) [file pgen.1003844.s006.docx]

Table S3. Primers used in this study.

| Name | Sequence 5'-3' | Use |
| --- | --- | --- |
| A10 | CTTTGTACTGATGATTTATATACTTCGGCATACGT | pWJ28 construct |
| AM1 | aaaaGTCGACATGATGCTTGAAATATAGTTGTG | pLM477 construct |
| B109 | TGTGCACCGGAACAGATGTATAG | 2537228^(a)^ |
| L106 | ggggACCACTTTGTACAAGAAAGCTGGGTTGCATGATTCAAATTGTTATTTGG | 2508018^(a)^ |
| L19 | aaaGGTACCTTTAAAGTATATATCAGATTGTTTCG | 2518543^(a)^ |
| L23 | CAAAGAGCTCGTCTACAAATTTC | 2513323^(a)^ |
| L340 | aaaGGGCCCTTTAAATACTTTTGTTGCCAATGTC | 2512967^(a)^ |
| L35 | TTTTGTATACAGGTGGTGGCC | 2515382^(a)^ |
| L50 | aaaAGATCTAATAATGTATTTACGCTGGGGC | 2518206^(a)^ |
| L6 | aaaGGTACCAAATTTAATGCTATTTTCCTTCGC | 2517502^(a)^ |
| L61 | GCCGAAGTATATAAATCATCAG | 2517804^(a)^ |
| L55 | TAAATCTAACAACACTCTAA | pWJ28 construct |
| L70 | aaaaAAGCTTCAAGAATCCAATGAAGTAGGGG | pG0400^(b)^ |
| L71 | aaaaAAGCTTCTAAATTAGAACATGATACTAACG | pG0400^(b)^ |
| L270 | aaaAAGCTTGTACAGATCTCGAGAGCTCCTAGGCTAGCATGCAAATAT  GAGCCAAATAAATATATTC | pLM9 construct |
| L271 | aaaGGTACCAGCTTCTGTAGGTTTTTAGGC | pLM9 construct |
| L299 | aaaGCTAGCAAATTTAATGCTATTTTCCTTCGC | pLM477 construct |
| T17 | GCGATGCTTCATATCGTGCG | 2510951^(a)^ |
| W10 | [CAATCAAACATCAGCCAATCGC](file:///C:\Users\Luciano%20Marraffini\AppData\Local\Microsoft\Windows\AppData\Roaming\Microsoft\Excel\Sequencing\IS256-like%20transposase.docx#OLE_LINK8 1,1546,1569,0,,CAATCAAACATCAGCCAATCGC) | IS*256*^(c)^ |
| W104 | TATGGTGATAAAGAGCAGATAAG | 2582417^(a)^ |
| W113 | AAATAACCTTTAACTATGGCTCC | 2431697^(a)^ |
| W115 | AGACACAGGTACTATGCCATC | 2438512^(a)^ |
| W117 | CTTTTGTTAAGGAGAGCAAAGC | 2435115^(a)^ |
| W12 | AAAAAATAATGATAATTGACGAGAC | 2514651^(a)^ |
| W121 | AAATTGGGGCATATTCTTGAACC | 2581387^(a)^ |
| W13 | TTATGGTTATTCAATTCTCAGATC | 2516985^(a)^ |
| W14 | ATCAATTTTTGTCCCAATTTTCAG | 2516186^(a)^ |
| W149 | TATCAAGTATTTCCTCATCATCG | 2521139^(a)^ |
| W15 | CAAATTACTGCTATATATTCAGGC | 2514570^(a)^ |
| W16 | TTAAATTTTATTATGAAGCAGGACG | 2513774^(a)^ |
| W161 | AACTTGTGTAATGAGTAAGCCTC | 2551795^(a)^ |
| W162 | TATGCTTATCGCCTTTGTCTCC | 2399121^(a)^ |
| W164 | TTCATCCCTAACACCCCTATC | 2469160^(a)^ |
| W17 | ACAAGAAACTGATTCAAGTGCTG | 2512540^(a)^ |
| W170 | TCTTCAGTTGTAATACTATTGGC | 2447577^(a)^ |
| W174 | TCGGAAGTCACTAGTTCCTTAG | 2358547^(a)^ |
| W177 | TTAATCCATAGTTGGCTGCTATG | 2553620^(a)^ |
| W179 | TGTCACTCCTCTACTGTATTATC | 2273662^(a)^ |
| W18 | TATTCTGAAAAGGTCAATCAAGG | 2511748^(a)^ |
| W19 | CTACTTTAATAATTGAAAAAGATGG | 2510135^(a)^ |
| W190 | AAAACCCCTGCATTTCTTCATTC | 2563690^(a)^ |
| W198 | TGATTCTAGTGAAAATCATCATAG | 2487258^(a)^ |
| W20 | GTCTTTTAAATATCAGAACAGTTAC | 2509349^(a)^ |
| W200 | TGTTAAAGTAGAAGGATCATCTG | 2492561^(a)^ |
| W203 | AAGGGCAATCCGAATACCACC | 2401456^(a)^ |
| W213 | TGTCATATTCAAATCCGTGCTG | 2495077^(a)^ |
| W233 | CTCTACTCACAAATTGCCAGTC | 2295234^(a)^ |
| W35 | GCATTAATTGCAACGATAACCG | 2494464^(a)^ |
| W69 | CTGATTAGGCAACAGTTATCCAG | 2584858^(a)^ |
| W9 | [CCATTAGTTGATTAAATACCGTTG](file:///C:\Users\Luciano%20Marraffini\AppData\Local\Microsoft\Windows\AppData\Roaming\Microsoft\Excel\Sequencing\IS256-like%20transposase.docx#OLE_LINK6 1,548,573,0,,CAACGGTATTTAATCAACTAATGG) | IS*256*^(c)^ |
| W94 | AGAAACAAGTTGGTGAGTATAAC | 2387024^(a)^ |
| W591 | ATACTCAACCAGTTTATGATTGTTCTCGTCCCCTTTTCTTC | pWJ87 construct |
| W592 | ACACTTCGTTATAGTAAATCTAACAACACTCTAAAAAATTG | pWJ87 construct |

^(a)^ Oligonucleotide used for primer walking. Numbers indicate the genomic coordinates of *S. epidermidis* RP62a where the 3’ end nucleotide of the primer anneals.

^(b)^ These primers amplify the *nickase* gene of the conjugative plasmid pG0400 and they were used to sequence *spc1* target site.

^(c)^ These primers anneal on the transposase gene of the IS*256* element and were used to sequence the site of insertion of escapers containing IS*256* insertions in the *cas* genes.
