# Supplementary material for: Dealing with the Evolutionary Downside of CRISPR Immunity: Bacteria and Beneficial Plasmids
Source: PLoS Genet. 2013 Sep 26;9(9):e1003844. doi: 10.1371/journal.pgen.1003844 (PMC3784566; doi:10.1371/journal.pgen.1003844)
Supplement: Text S1 — A Model for random generation of CRISPR-negative recipients and plasmid transfer. (DOCX) [file pgen.1003844.s007.docx]

**Supplementary Text 1. A Model for random generation of CRISPR-negative recipients and plasmid transfer.**

There are four populations of bacteria; CRISPR-positive recipients C_,_ CRISPR- negative recipients N, donors D, and Transconjugants T, CRISPR-negative recipients that have acquired the plasmid from the donors These variables C, N, D and T are both the densities these populations, bacteria per ml, and their designations. As in [[1](#_ENREF_1)], the rate of bacterial growth is proportional to the concentration of a limiting resource, *r* (µg/ml), respectively:

$$\psi_{C}(r)=\frac{v_{C}r}{(r+k)}, \psi_{N}(r)=\frac{v_{N}r}{(r+k)}, \psi_{D}(r)=\frac{v_{D}r}{(r+k)} and \psi_{T}(r)=\frac{v_{T}r}{(r+k)}$$

where *v*_C_, *v*_N_, *v*_D_ and *v*_T_ per cell per hour are the maximum growth rates of these bacterial populations and *k* the “Monod constant” (the concentration of the resource where bacterial growth is half its maximum value). The limiting resource is taken up at a rate proportional the densities of the bacteria, their rate of growth and a conversion efficiency parameter, *e* µg/ml per cell [[2](#_ENREF_2)]. Plasmid transfer is assumed to be a mass action process which occurs at a rate proportional to the product of the densities of plasmid-bearing cells (D+T), permissive CRISPR-negative recipients, *N*, and a rate constant, γ ml × cell/hr [[3](#_ENREF_3)] also see [[4](#_ENREF_4)]. CRISPR-positive cells cannot receive the plasmid. The rate of plasmid transfer declines and approaches 0 as the concentration of the limiting resource becomes depleted [[5](#_ENREF_5)].

With these definitions and assumptions, the rates of change in the density of the bacterial populations and the concentration of resource in a batch culture are given by:

$$\begin{matrix} \frac{dC}{dt}=\psi_{C}(r)C-\mu C \\ \frac{dN}{dt}=\psi_{N}(r)N+\mu C-\gamma\frac{r}{(r+k)}N(D+T) \\ \frac{dD}{dt}=\psi_{D}(r)D \\ \frac{dT}{dt}=\psi_{T}(r)T+\gamma N(D+T) \\ \frac{dr}{dt}=-e[\psi_{C}(r)C+\psi_{N}(r)N+\psi_{D}(r)D+\psi_{T}(r)T] \end{matrix}$$

where µ per cell per hour is the rate of mutation from C to N. We neglect mutation from N to C.

In our numerical solutions (computer simulations), population growth and plasmid transfer are deterministic processes but mutation from C to N is a stochastic. For the latter we use a Monte Carlo protocol and an Euler method for solving the differential equations with a step size, Δ*t*. When a random number (0<*x*<1) is less than the product NµΔtψ_N_(*r*) a single mutant enters the C population and is removed from the N. We chose values of Δ*t* such that NµΔtψ_N_(*r*)<1. Copies of the Berkeley Madonna (TM) program used for this simulation are available on www.eclf.net/programs.

To estimate the rate at which CRISPR-positive mutate to CRISPR-negative, µ (mutants per cell per hour) with this simulation we use parameter values in a range estimated in our experiments. We assume a conversion efficiency of *e*=5×10^-7^ µg per cell, a maximum resource concentration of 3750 µg/ml and growth rates of *S. aureus* donors and *S. epidermidis* recipients and transconjugants of 1.4 and 1.2 per cell per hour, respectively (which are in the range we estimate with a Bioscreen C). At time 0 in our simulations there are single donor and recipient cells (as there would be cultures initiated from single colonies). With these parameters and initial densities, by 17 hours the resource is exhausted and the densities of donors and recipients are in the range observed in our plasmid transfer experiments, 7.2×10^9^  and 2.8×10^8^, respectively. As our estimates of γ we use rate constants in the range estimated as Γ our plasmid transfer experiments in Table 1.

In the following figure we present of a single run with this simulation. Because of their higher growth rate, the donors D achieve a greater stationary phase density than the CRISPR-positive C population. As the density of C increases, CRISPR-negative mutants, N are produced and begin to grow. As the densities of these recipients and donors increase transconjugants T are generated and increase in density. Population growth and plasmid transfer ceases when limiting resource becomes depleted.


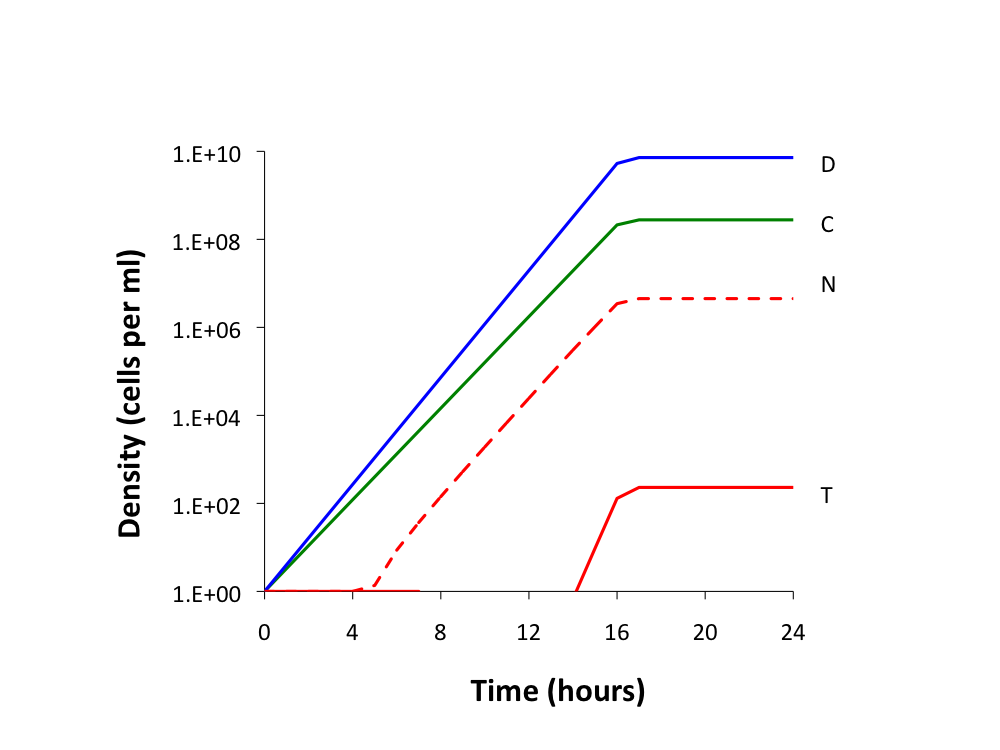


**Single simulation run.** Rate of change in the densities of donors (D), CRISPR-positive cells (C), CRISPR-negative mutants (N) that can receive the plasmid and transconjugants (T), CRISPR-negative cells that have received the plasmid. Parameters: *r* max= 3750 µg, *k*=1 µg/ml, *e*=5×10^‑7^µg, *v*_C_=*v*_N_=*v*_T_ =1.2 per hour, *v*_D_=1.4 per hour, µ=10^-3^ per cell per hour, γ=10^-14^ ml per cell per hour. Initial densities D=C=1 cell per ml and N=T=0 cells per ml.

Using these initial densities and different rate constants of plasmid transfer, we ran a series of simulations to estimate the value of µ needed obtain densities of transconjugants in the range observed in our fluctuation experiments, on the order of 150 per ml. With densities, growth and plasmid transfer rate parameters in the range seen in our experiments and CRISPR-loss mutation rates, µ, of between 10^-4^ and 10^-3^ provide mean and median numbers of transconjugants and variance/mean ratios similar to that observed in our fluctuation experiment (Table 2). The results of these simulations are described in the following table:

**Five independent simulations of 10 cultures with each parameter set.**

| **Mutation rate to CRISPR-negative µ** | **Plasmid transfer rate constant γ** | **Median** | **Mean** | **Variance/Mean Ratio** |
| --- | --- | --- | --- | --- |
| 10^-4^ | 10^-13^ | 166.9 | 321.1 | 409.8 |
|  |  | 116.1 | 168.3 | 141.0 |
|  |  | 114.6 | 182.1 | 80.5 |
|  |  | 134.7 | 216.2 | 219.5 |
|  |  | 141.0 | 187.8 | 90.0 |
| 5×10^-4^ | 2×10^-14^ | 133.8 | 134.9 | 6.8 |
|  |  | 134.5 | 149.3 | 10.1 |
|  |  | 141.2 | 281.4 | 299.4 |
|  |  | 210.3 | 309.1 | 293.2 |
|  |  | 112.1 | 215.1 | 431.4 |
| 10^-3^ | 10^-14^ | 134.6 | 140.2 | 5.3 |
|  |  | 126.6 | 156.5 | 42.7 |
|  |  | 154.5 | 167.1 | 17.6 |
|  |  | 145.4 | 203.3 | 183.1 |
|  |  | 147.9 | 381.8 | 1273.1 |

**Supplementary References.**

1. Monod J (1949) The growth of bacterial cultures. Annu Rev Microbiol 3: 371-394.

2. Stewart FM, Levin BR (1973) Resource partitioning and the outcome of interspecific competition: a model and some general considerations. Amer Nat 107: 171-198.

3. Stewart FM, Levin BR (1977) The Population Biology of Bacterial Plasmids: A priori Conditions for the Existence of Conjugationally Transmitted Factors. Genetics 87: 209-228.

4. Simonsen L (1990) Dynamics of plasmid transfer on surfaces. J Gen Microbiol 136 ( Pt 6): 1001-1007.

5. Levin BR, Stewart FM, Rice VA (1979) The kinetics of conjugative plasmid transmission: fit of a simple mass action model. Plasmid 2: 247-260.
