# Supplementary material for: Dealing with the Evolutionary Downside of CRISPR Immunity: Bacteria and Beneficial Plasmids
Source: PLoS Genet. 2013 Sep 26;9(9):e1003844. doi: 10.1371/journal.pgen.1003844 (PMC3784566; doi:10.1371/journal.pgen.1003844)
Supplement: Text S2 — A model for the population dynamics of conjugative plasmids in an immune CRISPR-positive population. (DOCX) [file pgen.1003844.s008.docx]

**Supplementary Text 2. A model for the population dynamics of conjugative plasmids in an immune CRISPR-positive population.**

As noted in the body of our report, in this model there two populations of recipients; CP - CRISPR-positive cells that can only receive plasmids bearing mutations in the spacer (CRISPR Escape Mutants (CEM), and CN – CRISPR – or spacer negative cells that can receive wild type or CEM plasmids. Donors and transconjugants are also each of two types; those bear the wild type plasmid, respectively, D1 and T1, and only transfer the plasmid to permissive recipients, CN, and D2 and T2 which bear the CEM plasmid and can transfer to the CP as well as the CN plasmid.

With a probability µ per cell per hour, CRISPR-positive cells lose a functional CRISPR-Cas system, CP🡪 CN. With a probability ν per cell per hour, CRISPR-escape mutant plasmids are generate, D1🡪D2. For simplicity and because the effects are negligible, we do not consider change in the states of the transconjugants, T1->T2, the acquisition of a functional CRISPR by CRIPR negative cells CN🡪CP or T2🡪T1 or loss of the plasmid by vegetative segregation. Plasmid transfer is a mass action process that occurs at a rate proportional to the product of the densities of donors and recipients and a rate constant, γ ml×cell×cell/hour [[1](#_ENREF_1),[2](#_ENREF_2)]. All six populations grow at the same rate which depends on the concentration of a limiting resource, *r* µg/ml $\psi(r)=\frac{Vr}{(k+r)}$where *V* is the maximum growth rate and the Monod constant, *k is the* concentration of the resource where the bacteria grow at half their maximum rate [[3](#_ENREF_3)]. Resource is consumed at a rate proportional to the rate of growth of the bacteria and a conversion efficiency parameter, *e* µg per cell [[4](#_ENREF_4)]. The rate of mutation and plasmid transfer declines as the resources are consumed so that when resources are fully consumed, and populations stop growing (are at stationary phase) neither mutants nor transconjugants are produced [[5](#_ENREF_5)].

With these definitions and assumptions, the rates of change in the densities of the bacterial populations and concentration of the limiting resource are given by,

*k* is the Monod constant, *v* the maximum growth rate and *e* the amount of the limiting resource necessary to produce a new cell.

For the numerical solution to these equations, population growth and plasmid transfer are deterministic processes, but mutation for the loss of CRISPR and the generation of mutant escape plasmids are stochastic. For the latter we use a Monte Carlo routine; when a random number *x* (0 < *x* <1) is less than the product of the mutation rates, µ or ν, density of the mutating population (CN or D1) and the step size Δ*t*, of the Euler method simulation, single mutants enter the CP or D2 population and are removed from the CN or D1 population.

**Supplementary References.**

1. Stewart FM, Levin BR (1977) The Population Biology of Bacterial Plasmids: A priori Conditions for the Existence of Conjugationally Transmitted Factors. Genetics 87: 209-228.

2. Simonsen L (1990) Dynamics of plasmid transfer on surfaces. J Gen Microbiol 136 ( Pt 6): 1001-1007.

3. Monod J (1949) The growth of bacterial cultures. Annu Rev Microbiol 3: 371-394.

4. Stewart FM, Levin BR (1973) Resource partitioning and the outcome of interspecific competition: a model and some general considerations. Amer Nat 107: 171-198.

5. Levin BR, Stewart FM, Rice VA (1979) The kinetics of conjugative plasmid transmission: fit of a simple mass action model. Plasmid 2: 247-260.
